# Supplementary material for: Virtual intracranial EEG signals reconstructed from MEG with potential for epilepsy surgery
Source: Nat Commun. 2022 Feb 22;13:994. doi: 10.1038/s41467-022-28640-x (PMC8863890; doi:10.1038/s41467-022-28640-x)
Supplement: Supplementary file 1 — Supplementary Information [file 41467_2022_28640_MOESM1_ESM.pdf]

Supplementary material

Network model

The derivation of functional connectivity and the subsequent mathematical modelling follows from multiple works looking for an optimal resection site using intracranial EEG [1, 2, 3]. In order to derive functional networks from the ViEEG time-series, 20 second epochs of seizure data containing clear ictal waveforms were chosen by MC, SV and CP. In the event that a seizure was shorter than 20s, the whole seizure was used. In the event that the entire recording showed semi-continuous ictal runs, epochs closer to the onset of the seizure were favoured. We then down-sampled the data to 512 Hz and band-pass filtered between 1 and 25 Hz using a 4th order Butterworth filter. We used these epochs to compute univariate iterated amplitude adjusted Fourier transform surrogates[4] for each epoch. We used 199 surrogates unless otherwise noted. Each epoch was then divided into 10 minimally overlapping sub-segments of 0.25 times the length of the original epoch. This resulted in 10 subsegments of the original time series and usually 1990 subsegments for the surrogates. We considered two functional connectivity methods: (1) Pearson correlation coefficient between amplitude envelopes (AEC) [5, 6] and (2) mutual information (MI) [1, 7, 8]. In order to accurately estimate the mutual information, we used the publicly available MILCA package [7]. To make the analyses computationally tractable, we reduced the number of surrogates to 19 when using mutual information to infer functional connectivity. We then used the Mann-Whitney-Wilcoxon U-test to assess whether the connections were significantly larger in the original time series (ρ_0) against the surrogate time series (ρ_{surr}). The surrogate-corrected connectivity matrix is as follows:

$$CC_{ij} = \frac{< \rho_{0_{ij}} > - < \rho_{surr_{ij}} >}{1 - < \rho_{surr_{ij}} >} h_{ij},$$

where $h_{ij} = 1$ if the null hypothesis of the statistical test was rejected, or $h_{ij} = 0$ otherwise. $< >$ indicates the median values over subsegments and surrogates.

We considered each of the nodes in each in ViEEG as connected neural masses using the theta model [2, 3]. The phase of each node follows the ODE:

$$\theta_j' = \left(1 - cos(\theta_j)\right) + \left(1 + cos(\theta_j)\right) I_j(t),$$

where the inputs are described by $I_j(t)$:

$$I_j(t) = I_0^j + \xi^j(t) + \frac{K}{N}\sum_{i \neq j} CC_{ij}\left(1 - cos(\theta_i - \theta_i^s)\right),$$

The index j denotes the node j, N is the total number of nodes, $I_0^j + \xi^j(t)$ is Gaussian noise, K is the global scaling parameter, a_{ij} is the $i, j - th$ entry in the functional connectivity network, and θ_i^S is the steady state of node i. Each node is initially in a 'normal state', which is a stable fixed point for the system, but can transition into the 'seizure state', a limit cycle, by passage through a saddle-node on invariant circle (SNIC) bifurcation.

As in Goodfellow et al.[1], we quantified the dynamics of the system using the notion of brain network ictogenicity BNI, which is the average fraction of time that each node spends in the 'seizure state'. To identify the seizure state, we transform the variable θ_i for node i using the function $T(\theta_i) = 0.5(1 - cos\,(\theta_i - \theta_i^s))$ which takes values in $[0, 1]$ where a value near 1 indicates a spike. Then, for this node, we identify points where $T > 0.9$. This marks the beginning of a node entering the seizure state. The node exits the seizure state if $T < 0.9$ for at least 24-time units ($\frac{24}{dt}$ time steps of the model), which indicates that no spikes have occurred. The *BNI* value is therefore obtained by computing the dynamical system over a long period of time (4×10^6 timesteps), with multiple runs to mitigate the effects of noise (128 noise runs) and averaging the time spent in the seizure state over all nodes, times, and runs. For a given network, the value of BNI will depend on the global scaling parameter, K. Therefore, for the full network, we find the value of K such that $BNI = 0.5$. We then use this value of K when simulating the surgical resection, as follows. In order to simulate surgical resection, we quantify the node ictogenicity (NI) of each node. To do this, we remove each node i individually and rerun the dynamical system to calculate a new BNI value, BNI_{post}^i. The value NI^i is then given by:

$$NI^i = \frac{BNI_{pre} - BNI_{post}^i}{BNI_{pre}},$$

where $BNI_{pre} = 0.5$. If BNI_{post}^i is small (aka $NI^{\,i}$ is large), then the node is considered ictogenic, or a candidate for resection, because removing it has the effect of reducing the fraction of time the dynamical system spends in the seizure state. Again, we run each of these

’virtual resections’ 128 times for 4 x10^6 timesteps. This generates a distribution of $NI^{\,i}$ values for each node removed. We then used a Mann-Whitney-Wilcoxon U-test and Bonferroni-Holms multiple comparisons correction to assess whether $NI^{\,i}$ is greater than the $NI^{\,i}$ over all removed nodes. The nodes i that have significantly large and positive NI^i become the VIZ.

Features of ictal ViEEG signals

We visually inspected each ViEEG seizure and ensured all ViEEG seizures analysed by dynamical network models have 1) visible transition from background activity to ictal waveforms that is aligned in time with seizure onset annotated by C.P. using MEG sensor signals and 2) distinctive morphological features and spatial distributions of ictal waveforms that can resemble seizures recorded by iEEG, if iEEG is done (Fig. 2, Supplementary Fig. 17). Example ictal ViEEG signals from each seizure are presented in Supplementary Figs. 5-16 and comparison between iEEG and ViEEG total sensor counts are shown in Supplementary Table 1. Ictal ViEEG signals were plotted in a 10-second window using seizure onset as time zero and 2 seconds before seizure onset and 8 seconds after seizure onset. As shown in Fig. 2 (main paper), ictal ViEEG signals present distinctive ictal rhythms akin to an ictal event independently recorded by iEEG. This case also supports the previous studies that MEG can 'see' activities from deep structures [9, 10, 11, 12] and seizures can be reconstructed from those sources with similar features to an iEEG seizure.

As discussed in the main paper, not all MEG seizures were reconstructed with clear ictal waveforms or a distinct transition to seizure state. An example is given in Supplementary Fig 1, where the second MEG seizure from Patient 5 (ictal ViEEG signals reconstructed from the first MEG seizure is shown in Supplementary Fig. 9) were reconstructed using ViEEG but did not present clear ictal waveforms or a distinct transition to the seizure state.

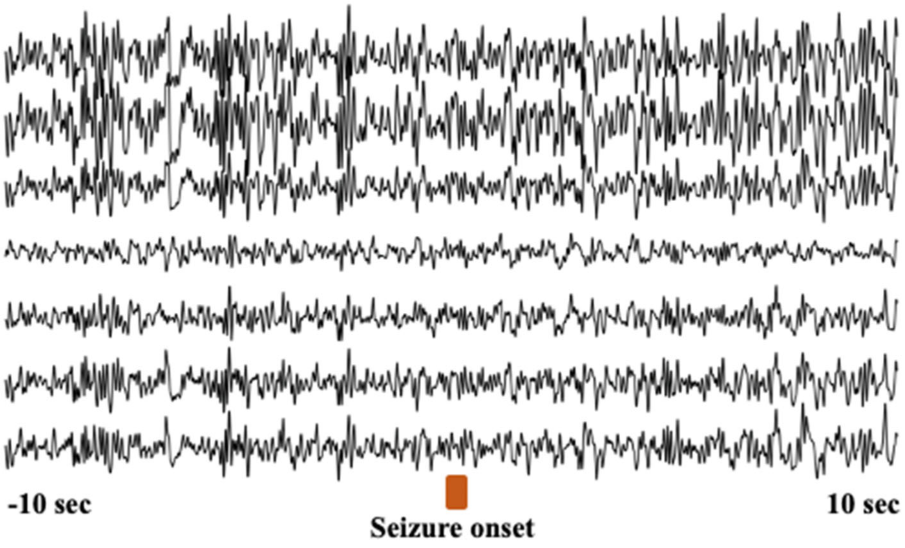

Supplementary Figure 1. An example of reconstructed ictal ViEEG signals (seizure 2) from Patient 5 (the same ViEEG electrodes were used to plot the signals) that do not present distinct morphological features.

Considerations for ViEEG locations

iEEG locations play a critical role in clinically characterising the EZ. Therefore, whether ViEEG locations affect our proposed model to characterise the EZ non-invasively is an important question to ask. As discussed in the main paper, ViEEG locations are defined to extensively cover the early-, mid-, and late-phase MSL solutions as well as the entirety of the resection margin. It is important to note that the choice of location, shape and orientation of ViEEG does not take into account any other information (such as shape of resection or pathology) – they are defined in a non-regularised fashion to sufficiently sample the targeted brain areas along the cortical surface (like subdural grid electrodes) and linearly (like stereotactic depth electrodes) along deep cortical structures, such as hippocampal structures. As well, ViEEG and network models do not require the resection margin and MSL solutions to be in any specific locations within the ViEEG, such as the centre of the ViEEG. When defining the ViEEG, we always ensure there is sufficient brain tissue between the ViEEG boundary and the boundary of resection and MSL solutions. In Patient 5 (Supplementary Fig. 2A), we demonstrate the same AEC-VIZ 'hotspot' location is found by two different ViEEGs modelled independently. ViEEG1 and ViEEG2 grid and depth electrode set-ups differ but the resulting left baso-mesial temporal VIZ localisations are similar. Providing the area of interest is covered for the VIZ analysis, these results suggest our proposed model is less likely to be affected by how the ViEEG is initially defined. A second example is given by Patient 11 (Supplementary Fig. 2B), where a 4-by-4 grid like ViEEG is defined at the contralateral temporal area (less clinically concerning) together with a 10-by-10 grid covering the resection margin and MSL locations. The additional 16 nodes from the 4-by-4 grid covering a contralateral brain area does not affect the model in identifying the VIZ hotspot location that is concordant with the iEEG SOZ. To ensure that ViEEG signals represent the actual neural dynamics, all ViEEG electrodes are defined inside inner-skull surface and mostly in grey matter. An example of locations of ViEEG electrodes is shown in Supplementary Fig 2C with ViEEG electrode locations plotted on brain model and MR images.

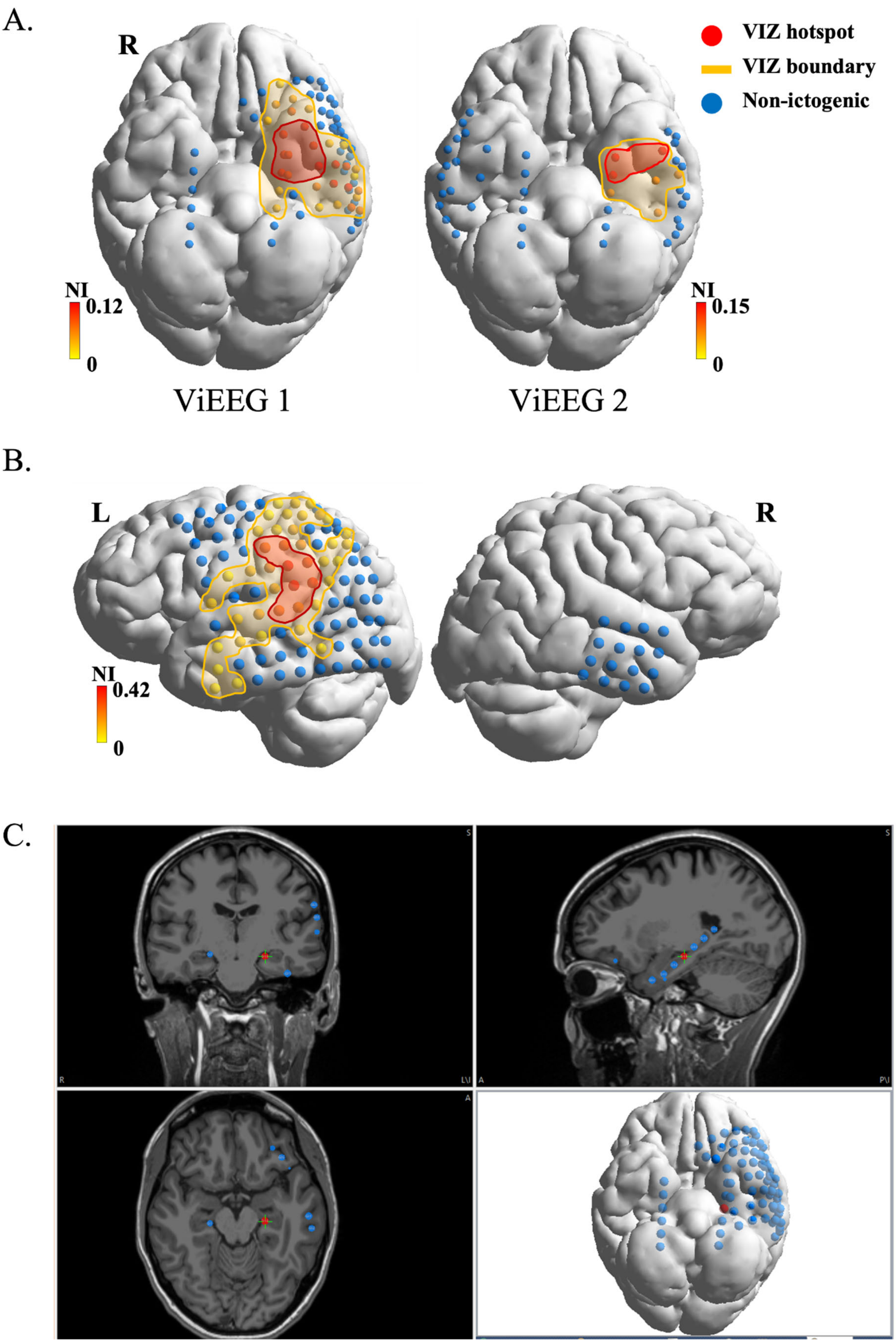
A.
R
VIZ hotspot
VIZ boundary
Non-ictogenic
NI
0.12
0
NI
0.15
0
ViEEG 1
ViEEG 2
B.
L
R
NI
0.42
0
C.

Supplementary Figure 2. Variation in the initial ViEEG set-up appears to have minimal effect on the VIZ result, providing the area of interest is covered. The VIZ for Patient 5 (Supplementary Fig. 2A) remains left temporal baso-mesial despite differences in the initial ViEEG configuration. The VIZ for Patient 11 (Supplementary Fig. 2B) is unaffected by the introduction of a contralateral (right) 16-electrode ViEEG grid. VIZ boundary and hotspot results have been derived from the AEC-VIZ method. When defining ViEEG, locations of ViEEG electrodes are ensured to lie inside the inner-skull surface and predominantly in the cortical grey matter involving both gyral and sulcal surfaces. An example of ViEEG electrode locations is demonstrated here (Supplementary Fig. 2C) with brain model (transparency 50%) and MR images. For ease of orientation, a red-coloured ViEEG left hippocampal depth electrode in the brain model figure (Supplementary Fig 2C right lower panel) is also coloured in red in the MR images (Supplementary Fig 2C left panel, right upper panel). Because all cortical reconstructions are rendered 50% transparent to permit visualisation of the ViEEG electrodes, they are actually deeper than they appear – here they sit within the bed of the cortical ribbon (left lateral temporal grid) and in the deep grey matter (bilateral hippocampal depths) and not superficially at the dural surface) .

Abbreviations: ViEEG = virtual intracranial electroencephalography, VIZ = virtual ictogenic zone, AEC = amplitude envelope correlation, MI = mutual information, NI = node ictogenicity

Patient ID	Number of iEEG electrodes	Number of ViEEG electrodes
1	64	145
2	iEEG not done	176
3	iEEG not done	81
4	40	160
5	40	76
6	iEEG not done	164
7	Single intraoperative depth	96

8	iEEG not done	208
9	64	64
10	64	132
11	56	116
12	64	100

Supplementary Table 1. Number of iEEG electrodes in the eight patients who underwent iEEG and the number of ViEEG electrodes for all patients.

Abbreviations: iEEG = intracranial electroencephalography , ViEEG = virtual intracranial electroencephalography

ViEEG signal reconstruction

We defined ViEEG electrodes to cover brain areas that have been source localised using MEG (early, mid and late) from the previous publication [11] and ensured to contain the entire resection bed with sufficient margin between the boundaries of resection and ViEEG electrodes. In other words, ViEEG was only guided by MEG source localisation and resection margins (and not the iEEG array or other clinical information). Next, we attempted to reconstruct ictal source signals of each ViEEG electrode akin to what is recorded invasively with iEEG. Bad MEG channels were identified and omitted from raw MEG recordings during and after data acquisition. A temporal extension to signal source separation (tSSS) was then applied to MEG sensor signals using *Maxfilter v2.2.10-15* (Elekta Oy) for interference suppression. After tSSS, a notch filter was applied to remove line noise at 50 Hz and its harmonics up to 300 Hz and an IIR filter to bandpass filter signals between 0.1 and 200 Hz.

Pre-processed MEG signals were then segmented into epochs of 10 minutes before seizure onset and the whole seizure event from seizure onset to seizure termination. Onset and offset of each seizure were annotated by C.P. and also reported in the previous publication [11]. Long epochs and broad frequency bands were used for source reconstruction to more reliably estimate noise covariance matrices and alleviate the suppression of correlated sources by beamformer techniques [13, 14]. Empirically, we also found shorter epochs often resulted in less distinctive morphologies and more smeared spatial distributions of ictal source signals.

A scalar beamformer technique was employed to reconstruct ictal ViEEG signals [15]. The orientation of each dipolar source was computed to maximise source power [16, 17]. Beamformer techniques have been used to successfully reconstruct source signals for various applications at high spatial resolution, particularly in the context of MEG virtual electrodes [17, 18]. More specifically, given a ViEEG electrode, we constructed a set of beamformer weights that spatially filter source activity at this location without contribution from other sources. We used an implementation of linearly constrained minimum variance (LCMV) beamformer with orientations optimised by maximal source power from MNE-Python Version 0.19.0 [19]. To construct a beamformer at each ViEEG electrode, the data covariance matrix was estimated using the whole seizure event (from seizure onset to seizure termination), while the noise covariance matrix was estimated using a pre-seizure segment (i.e., -600 second to -10 second

if seizure onset is defined as 0 second). The pre-seizure segment had been visually inspected to ensure no ictal activity is included. We used realistic boundary element method (BEM) models generated from individualised MRI scans to compute forward solutions. Triangulated mesh surfaces of inner-skull and pial surface were generated using the patient-specific MRI scan, Freesurfer [20] and CURRY 8® (Compumedics Neuroscan®, Hamburg) software. After source signal reconstruction, we visually inspected ictal ViEEG signals and identified 25/36 seizures from 12 patients that presented a distinctive morphology and spatial distribution from background activity. These 25 seizures were then analysed by dynamical network models.

Volume conduction and functional networks

Volume conduction introduces source signal leakage that affects source signal reconstruction and structures of time-evolving EEG and MEG source networks. We explored extensively in parameter space to optimise the spatial resolution and signal strength of ictal ViEEG signals. However, these efforts are not guaranteed to completely remove spurious interactions from MEG source networks. Palva et al.[4] using simulations and realistic head models demonstrated that the currently available methods cannot completely remove spurious connections. In other words, when there is a true connection, spurious connections always accompany. Another study from Hincapie et al.[20] suggests different source reconstruction techniques, size and locations of correlated sources also change the extent to which field leakage impacts source signals. However, a limitation of both studies is that only two genuinely correlated sources were taken into account in their simulations [5, 21]. Moreover, the connectivity methods used to reduce instantaneous phase synchrony may have been too conservative to preserve important network structures, particularly if more than two sources genuinely correlate. For iEEG studies and our proposed ViEEG approach, the assumption of only two correlated sources in the network is less likely to be valid when a seizure occurs.

Because we aimed to explore clinical biomarkers that pre-surgically characterise the EZ in a non-invasive fashion, the connectivity methods we employed did not remove instantaneous spurious connections in an effort to better preserve key functional network structures [22]. Note also that we did not attempt to interpret our findings in the context of neural mechanisms related to seizure generation. Biomarkers and limitations are discussed with the support of statistical analysis (main paper and below).

Different thresholds to define the VIZ hotspot

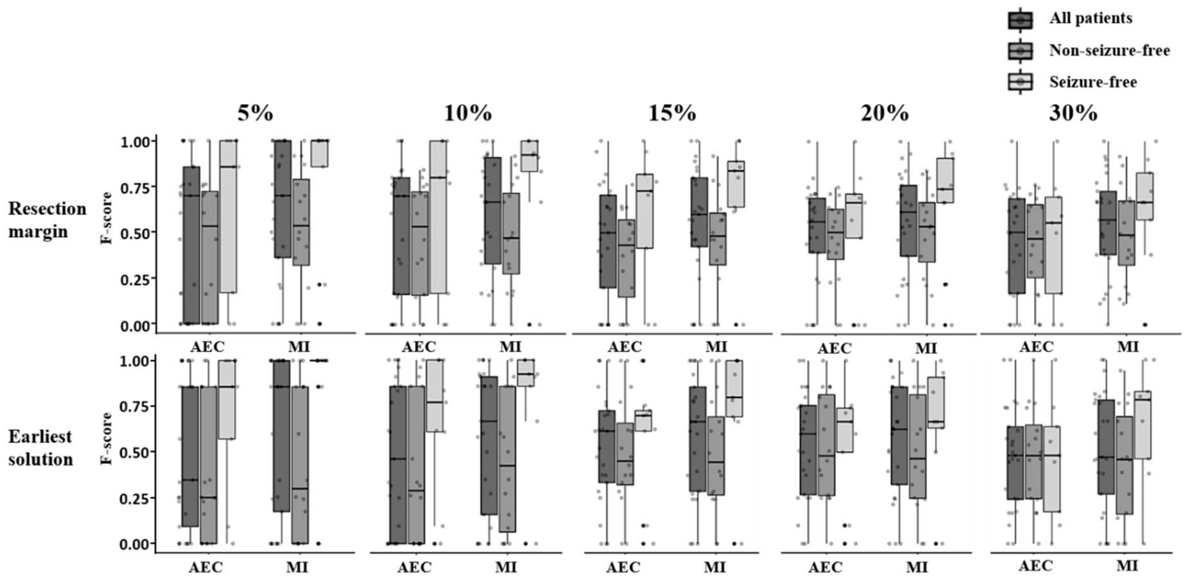

Supplementary Fig. 3. F-scores of AEC-VIZ and MI-VIZ in predicting the resection margin and the earliest solution using different thresholds to define the VIZ hotspot.

An additional four thresholds, 5%, 10%, 15% and 30%, were explored along with 20% threshold to define the VIZ hotspot through ranking all VIZ nodes by NI values. Although MI-VIZ achieve remarkable F-scores to predict the resection margin and the earliest solution, the top 5% and 10% thresholds do not fully represent the predictive power of the models, as too few VIZ nodes are defined as hotspot sources. For example, the MI-VIZ from Patient 1 Seizure 1 (Supplementary Fig. 5) has 34 VIZ nodes, which results in two and three hotspot nodes respectively if 5% and 10% thresholds are applied. When thresholds are over 10%, such as 15%, 20% and 30%, F-scores of AEC-VIZ and MI-VIZ are relatively similar. Although 15% threshold seems to offer the optimal predictive power among five thresholds we explored, in this paper we presented results from the top 20% threshold of VIZ nodes to be defined as hotspots to ensure that our work has the same thresholding strategy that was used by HDEEG and MEG source localisation in our previous publication[11]. This threshold accommodates source localisation probability map sLORETA maxima without excessive smearing of the solution at lower thresholds[23], which can lead to an overestimation of the accuracy of results based on the degree of overlap with the iEEG localisation and resection margins. Optimal thresholding for ViEEG requires further exploration in a prospective study when 'virtual' resection margins are defined before surgery. Note that Odds Ratios, Precision,

and Recall are based on n=25 seizures with n=9 seizures (from seizure-free group of 6 patients) and n=16 seizures (from non-seizure-free group of 6 patients). Boxplot minima (minimum data value), maxima (maximum data value), lower boundary (25th percentile), upper boundary (75th percentile); Whisker lower boundary (25th percentile minus 1.5 times interquartile range Q3-Q1) , upper boundary (75th percentile plus 1.5 times interquartile range Q3-Q1). Black dot refers to outlier.

Abbreviations: VIZ = virtual ictogenic zone, AEC = amplitude envelope correlation, MI = mutual information

Statistical analysis

First, we evaluated whether there is any association between VIZ hotspots and boundaries against the clinical localisation. Mixed-effects logistic regression modelling was used, with the outcome being resection margin, iEEG SOZ, early-MSL, mid-MSL, late-MSL, and the earliest solution (whether given by early-MSL or early-ESL). The variable in the modelling was a binary variable with 1 if a node was in VIZ hotspot or VIZ boundary and 0 if a node was not in VIZ hotspot or VIZ boundary. Results are expressed as odds ratios (OR) with 95% confidence interval (CI), p-values, Akaike Information Criterion (AIC) [24] and Bayesian Information Criterion (BIC) [25] (Supplementary Table 2, Supplementary Table 3). Both BIC and AIC were used to compare AEC and MI methods for the logistic regression.

	**AEC-VIZ hotspot**				**MI-VIZ hotspot**			
**Outcome**	**OR (95% CI)**	**AIC**	**BIC**	**P-value**	**OR (95% CI)**	**AIC**	**BIC**	**P-value**
Resection margin	4.701 (3.185, 6.595)	1825	1843.2	<0.001	7.232 (4.853, 10.56)	1773.5	1791.7	<0.001
iEEG SOZ	6.758 (3.225, 9.902)	599.1	617.4	<0.001	5.268 (2.101, 9.219)	615.4	633.6	<0.001
Earliest solution	4.158 (2.738, 6.147)	1451.1	1469.4	<0.001	5.658 (3.891, 8.139)	1431.7	1449.9	<0.001
Early-MSL	3.617 (2.4, 5.326)	1373.5	1391.8	<0.001	3.786 (2.495, 5.74)	1365.2	1383.4	<0.001
Mid-MSL	1.492 (0.644, 2.011)	1423.7	1441.9	0.061	1.847 (1.057, 2.927)	1427.3	1445.5	0.002
Late-MSL	1.08 (0.556, 1.917)	1216.8	1235.1	0.802	1.452 (0.797, 2.47)	1215.3	1233.5	0.185

Supplementary Table 2. Odds ratios (95% CI), AIC, BIC and 2-sided p-values from mixed-effect logistic regression model of relationship between VIZ hotspot and clinical localisation (resection margin, iEEG SOZ, early-MSL, mid-MSL, late-MSL, and the earliest solution). No adjustments were made for multiple comparisons.

Abbreviations: iEEG intracranial EEG, SOZ = seizure onset zone, VIZ = virtual ictogenic zone, MSL = MEG source localisation, ESL = HDEEG source localisation, AEC = amplitude envelope correlation, MI = mutual information.

	AEC-VIZ boundary				MI-VIZ boundary			
**Outcome**	**OR (95% CI)**	**AIC**	**BIC**	**P-value**	**OR (95% CI)**	**AIC**	**BIC**	**P-value**
Resection margin	2.52 (2.02, 3.14)	2261.8	2280.2	<0.001	4.67 (3.70, 5.91)	2151.6	2170	<0.001
iEEG SOZ	3.04 (1.95, 4.75)	648.55	664.66	<0.001	3.74 (2.37, 5.89)	639.5	655.6	<0.001
Earliest solution	3.22 (2.47, 4.18)	1784.5	1802.9	<0.001	3.51 (2.70, 4.57)	1729.9	1748.3	<0.001
Early-MSL	1.42 (1.06, 1.91)	1734.67	1753.1	<0.001	1.59 (1.18, 2.13)	1723	1741.4	<0.001
Mid-MSL	1.11 (0.81, 1.54)	1482.9	1501.3	0.02	1.44 (1.04, 1.98)	1479.2	1479.6	0.002
Late-MSL	3.19 (2.46, 4.13)	1314	1332.4	0.518	4.61 (3.53, 6.03)	1309.6	1328	0.027

Supplementary Table 3. Odds ratios (95% CI), AIC, BIC and p-values from mixed-effect logistic regression model of assessing statistical relationship between VIZ boundary and clinical localisation (resection margin, iEEG SOZ, early-MSL, mid-MSL, late-MSL, and the earliest solution). No adjustments were made for multiple comparisons.

Abbreviations: iEEG intracranial EEG, SOZ = seizure onset zone, VIZ = virtual ictogenic zone, MSL = MEG source localisation, ESL = HDEEG source localisation, AEC = amplitude envelope correlation, MI = mutual information.

Next, we calculated precision (or positive predictive values) and recall (or sensitivity) of VIZ hotspot and VIZ boundary for predicting the resection margin and clinical localisation. The precision of VIZ hotspot and recall of VIZ boundary were used to compute F-scores to assess the performance of our proposed model in predicting the clinical localisation (Supplementary Fig. 4).

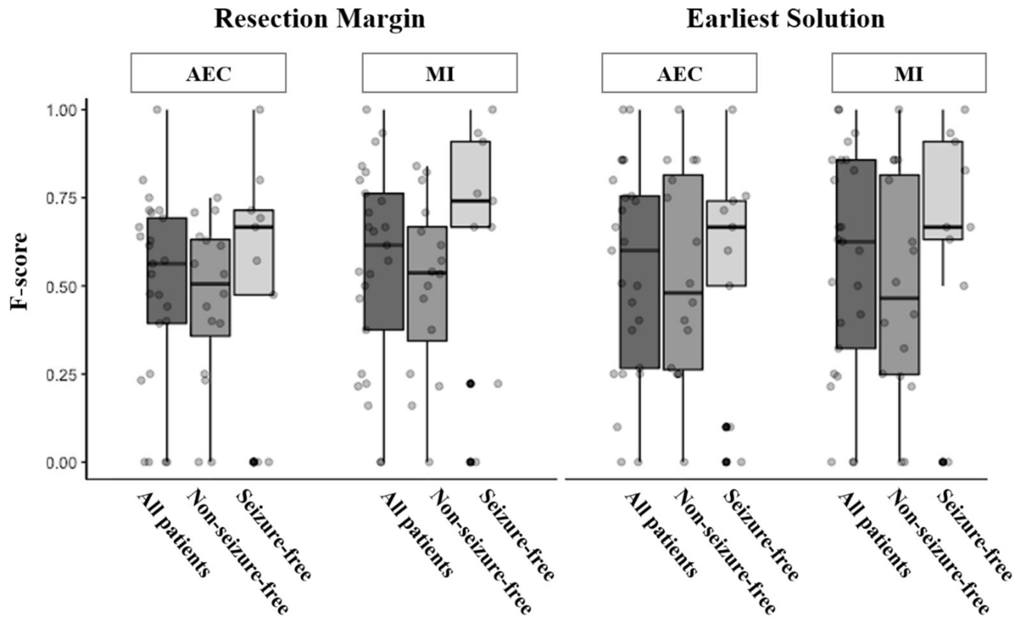

Supplementary Figure 4.

F-scores for AEC-VIZ and MI-VIZ predicting the resection margin and earliest solution are presented in boxplots (horizontal bar, box upper boundary, box lower boundary and dots represent median, first and third quartile and each individual VIZ respectively). MI-VIZ demonstrates higher F-scores (median 0.75) than AEC-VIZ (median 0.68) in predicting the EZ (i.e., resection margin from seizure-free patients). AEC-VIZ and MI-VIZ do not show a difference between seizure-free and non-seizure-free groups in predicting the earliest solution. Note that Odds Ratios, Precision, and Recall are based on n=25 seizures with n=9 seizures (from seizure-free group of 6 patients) and n=16 seizures (from non-seizure-free group of 6 patients). Boxplot minima (minimum data value), maxima (maximum data value), lower boundary (25th percentile), upper boundary (75th percentile); Whisker lower boundary (25th percentile minus 1.5 times interquartile range Q3-Q1), upper boundary (75th percentile plus 1.5 times interquartile range Q3-Q1). Black dot refers to outlier. No adjustments were made for multiple comparisons.

Abbreviations: AEC = amplitude envelope correlation, MI = mutual information.

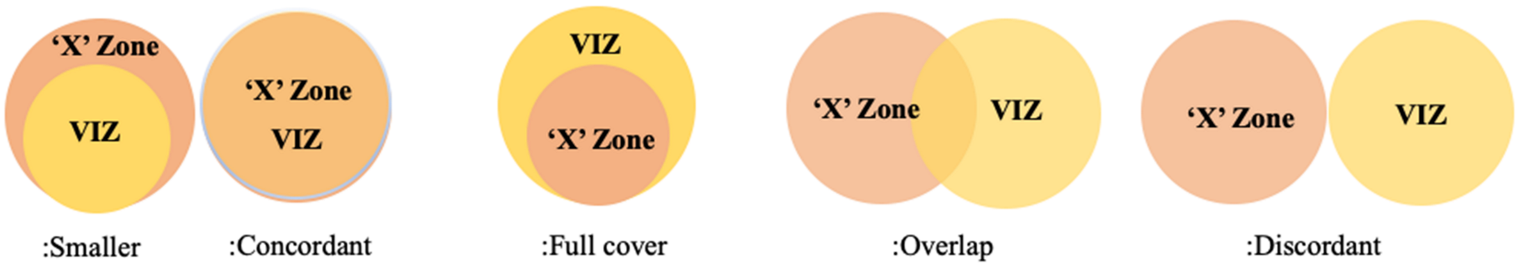

'X' zone	Outcome	Patient	seizure		Patient	seizure		Patient	seizure		Patient	seizure	
			AEC	MI		AEC	MI		AEC	MI		AEC	MI
Resection margin	Seizure-free	-/6	-/9	-/9	4	6	8	1	1	0	1	2	1
	Non-seizure-free	-/6	-/16	-/16	1	2	2	4	8	10	1	6	4
iEEG SOZ		-/2	-/3	-/3	1	1	3	1	1	-	-	1	-
		-/4	-/8	-/8	2	3	3	1	1	1	1	4	4
Earliest solution		-/6	-/9	-/9	4	6	8	1	1	-	1	2	1
		-/6	-/16	-/16	2	5	4	2	5	6	2	6	6
Early-MSL		-/6	-/9	-/9	3	4	6	1	1	-	2	4	3
		-/6	-/16	-/16	2	4	4	2	5	6	2	7	6
Mid, late - MSL		-/6	-/9	-/9	2	2	2	-	-	-	4	7	7
		-/6	-/16	-/16	1	5	5	2	3	3	3	8	8

Supplementary Table 4. Per-patient and per-seizure concordance between VIZ (AEC and MI) and clinical localisation. The five categories of concordance (at top) are based on spatial overlap relations between the VIZ boundary (VIZ) and clinical localisation ('X' zone), and are given as smaller VIZ, concordant, VIZ fully covers X zone, VIZ and X zone overlap, discordant. X zones (at left) are given as resection margin, iEEG SOZ, MSL solutions (early-, mid-, late-MSL) and the earliest source localisation solution. Patients and seizures are grouped based on surgical outcome (seizure-free or non-seizure-free). No VIZ boundary is found to be smaller or completely concordant with any X zone.

Abbreviations: iEEG = intracranial electroencephalography, SOZ = seizure onset zone, VIZ = virtual ictogenic zone MSL = MEG source localisation, AEC = amplitude envelope correlation, MI = mutual information

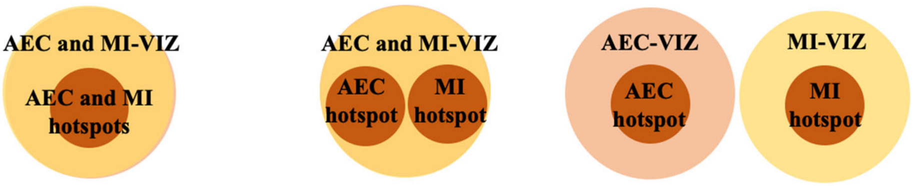

Outcome	Boundary concordant with the same 'hotspot'/seizure	Boundary concordant with the different 'hotspot'/seizure	Boundary discordant
Seizure-free	7/9	-	2
Non-seizure-free	9/16	1	6

Supplementary Table 5. Spatial overlap between respective hotspots and boundaries for AEC-VIZ and MI-VIZ . Seizure counts are grouped based on surgical outcomes (seizure-free and non-seizure-free). 16/25 seizures have AEC-VIZ hotspot and boundary concordance with MI-VIZ, while 9 seizures have AEC-VIZ with a different hotspot from MI-VIZ, with the majority of these (8/9 seizures) discordant for the VIZ boundary as well.

Abbreviations: iEEG = intracranial electroencephalography, SOZ = seizure onset zone, VIZ = virtual ictogenic zone, MSL = MEG source localisation, AEC = amplitude envelope correlation, MI = mutual information

It is also worth noting that AEC-VIZ and MI-VIZ do not always present concordant hotspots or boundaries. As shown in Supplementary Table 5, 16/25 seizures show AEC-VIZ and MI-VIZ concordance for both hotspot and boundary, 9/25 seizures show AEC-VIZ and MI-VIZ discordance for hotspot, with the majority of these patients experiencing seizure recurrence post-operatively. Thus, discordance of AEC-VIZ and MI-VIZ hotspots may offer complementary information and additional insights to alternative surgical strategies for non-seizure-free patients.

Patient ID	MRI	Histology	Surgical outcome	VIZ vs Resection margin			VIZ vs iEEG SOZ			VIZ vs Early-MSL			VIZ vs Earliest solution			
				Seizure	AEC	MI	Seizure	AEC	MI	Seizure	AEC	MI	Earliest solution	Seizure	AEC	MI
1	Normal	CD 1A	Engel class I<br>Rare NDS	1	PO	PO	1	PO	PO	1	FC	PO	Early-MSL	1	FC	PO
2	Normal	CD 1	Engel class I<br>Seizure free	1	FC	FC	iEEG is not done			1	FC	FC	Early-ESL	1	FC	FC
				2	FC	FC				2	FC	FC		2	FC	FC
3	Normal	CD 2A	Engel class I<br>Rare NDS	1	PO	PO	iEEG is not done			1	PO	PO	Early-MSL	1	PO	PO
				2	PO	PO				2	PO	PO		2	PO	PO
				3	PO	PO				3	PO	PO		3	PO	PO
				4	PO	PO				4	PO	PO		4	PO	PO
				5	NO	PO				5	FC	FC		5	FC	FC
				6	PO	PO				6	PO	PO		6	PO	PO
4	Normal	Non-specific	Engel class III<br>Fewer DS	1	NO	NO	1	NO	NO	1	NO	NO	Early-MSL	1	NO	NO
				2	NO	NO	2	NO	NO	2	NO	NO		2	NO	NO
				3	NO	NO	3	PO	PO	3	NO	NO		3	NO	NO
				4	FC	FC	4	FC	FC	4	NO	NO		4	NO	NO
				5	NO	NO	5	NO	NO	5	NO	NO		5	NO	NO
5	Normal	CD 1C	Engel class I<br>Seizure free	1	FC	FC	1	FC	FC	1	FC	FC	Early-MSL	1	FC	FC
6	Normal	CD 2A	Engel class I<br>Seizure free	1	FC	FC	iEEG is not done			1	FC	FC	Early-ESL	1	FC	FC
7	Normal	CD 2A	Engel class I<br>Seizure free	1	NO	NO	Only iEEG report available*			1	NO	NO	Non-localising	1	NO	NO
8	Normal	CD 1	Engel class I<br>Seizure free	1	FC	FC	iEEG is not done			1	NO	NO	Early-ESL	1	FC	FC
				2	FC	FC				2	NO	NO		2	FC	FC

Patient ID	MRI	Histology	Surgical outcome	VIZ vs Resection margin			VIZ vs iEEG SOZ			VIZ vs Early-MSL			VIZ vs Earliest solution			
				**Seizure**	**AEC**	**MI**	**Seizure**	**AEC**	**MI**	**Seizure**	**AEC**	**MI**	**Earliest solution**	**Seizure**	**AEC**	**MI**
9	Normal	CD 2B	Engel class II Rare DS	1	PO	PO	Only iEEG report available*			1	NO	NO	Early-ESL	1	NO	NO
10	Multi-lobar dysplasia	Normal	Engel class I NDS only	1	FC	FC	1	FC	FC	1	FC	FC	Early-MSL	1	FC	FC
11	Multi-lobar dysplasia	Ischemia	Engel class III Fewer DS	1	NO	PO	1	FC	FC	1	FC	FC	Early-MSL	1	FC	FC
				2	PO	PO	2	FC	FC	2	FC	FC		2	FC	FC
12	Right frontal gliosis	Gliosis	Engel class I Seizure free	1	PO	FC	1	PO	FC	1	PO	FC	Early-ESL	1	PO	FC
				2	NO	FC	2	NO	FC	2	NO	FC		2	NO	FC

Supplementary Table 6. Patient data and overlap with VIZ results for all seizures. *Only iEEG report available for patient 7 and 9.

Abbreviations: NDS = non-disabling seizures, DS = disabling seizures, FC = Full Cover, PO = Partial Overlap; NO = No Overlap, VIZ = virtual ictogenic zone, iEEG SOZ = iEEG seizure onset zone, MSL = magnetoencephalographic source localisation, CD = cortical dysplasia,

Results Summary (also refer to Supplementary Table 6)

Patient 1

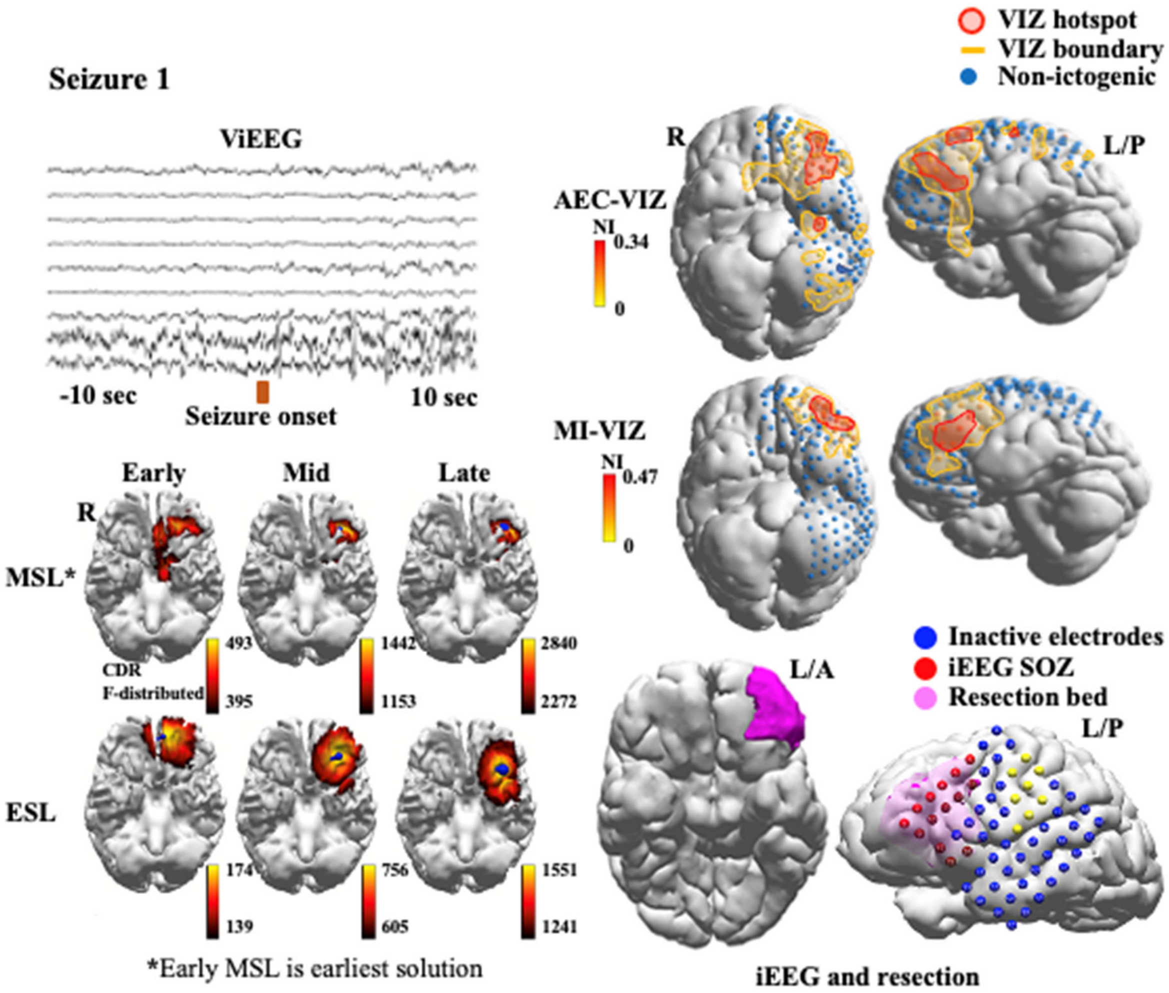

Supplementary Figure 5. Patient 1 had a normal MRI with over 10 disabling seizures per month before surgery. The patient had five disabling seizures when medication was adjusted but is now seizure-free again at month 39. MEG sLORETA ictal early source localisation suggested a focus at the left medial orbitofrontal gyrus and rectal gyrus. Guided by MSL solutions, ViEEGs were defined to extensively cover the left orbitofrontal, lateral frontal, and temporo-parietal areas. Source signals of six seizure events were reconstructed but only the first seizure gave a distinct morphology for ictal spikes. AEC-VIZ and MI-VIZ identified hotspot (nodes in red line and shade) encompassing two isolated islands: one in the orbitofrontal and the other in dorsolateral frontal convexity. The orbitofrontal component of

AEC-VIZ and MI-VIZ best overlap the MSL sLORETA solutions and partially overlap the surgical resection bed. This patient has achieved Engel I outcome with rare non-disabling seizures. Based on the surgical outcome, the MEG derived AEC-VIZ and MI-VIZ results suggest another epileptic focus outside the current resection volume.

Abbreviations: MEG = magnetoencephalography, iEEG = intracranial electroencephalography , ViEEG =virtual intracranial electroencephalography , EZ = epileptogenic zone, SOZ = seizure onset zone, HDEEG =high density electroencephalography, VIZ = virtual ictogenic zone, MSL = MEG source localisation, ESL =HDEEG source localisation, AEC = amplitude envelope correlation, MI = mutual information, AEC-VIZ =virtual ictogenic zone using amplitude envelope correlation, MI-VIZ = virtual ictogenic zone using mutual information, NI = node ictogenicity.

Patient 2

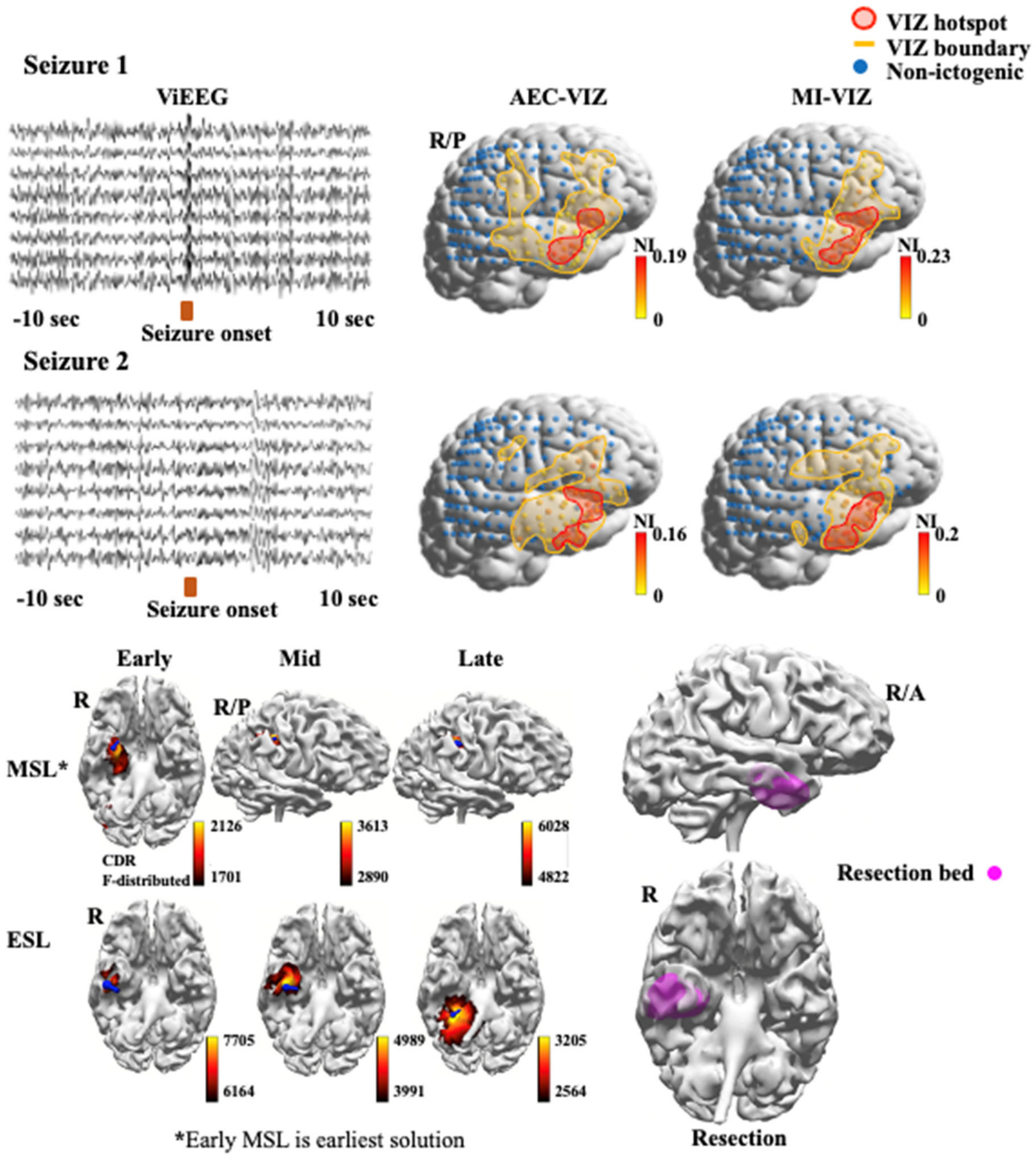

Supplementary Figure 6. Patient 2 had a normal MRI with an average of 5 seizures per month before surgery and is seizure free at 26 months follow-up. MEG sLORETA ictal early source localisation suggested a focus at right temporal pole. Guided by MSL solutions, ViEEG was defined to extensively cover the right temporal pole, lateral temporal, parietal, and lateral frontal areas. Source signals of two MEG captured seizure events are reconstructed. Ictal discharges can be seen in the representative ViEEG channels. Both AEC-

VIZ and MI-VIZ identified hotspots (nodes in red line and shade) over the right lateral temporal pole. Note the overlap with the early MSL solution and the surgical resection and the lack of overlap with the ESL solutions, which were more postero-basal in location. This patient has achieved an Engel I seizure-free outcome. Both the AEC-VIZ and MI-VIZ successfully captured the putative EZ.

Abbreviations: MEG = magnetoencephalography, iEEG = intracranial electroencephalography , ViEEG =virtual intracranial electroencephalography , EZ = epileptogenic zone, SOZ = seizure onset zone, HDEEG =high density electroencephalography, VIZ = virtual ictogenic zone, MSL = MEG source localisation, ESL =HDEEG source localisation, AEC = amplitude envelope correlation, MI = mutual information, AEC-VIZ =virtual ictogenic zone using amplitude envelope correlation, MI-VIZ = virtual ictogenic zone using mutual information, NI = node ictogenicity.

Patient 3

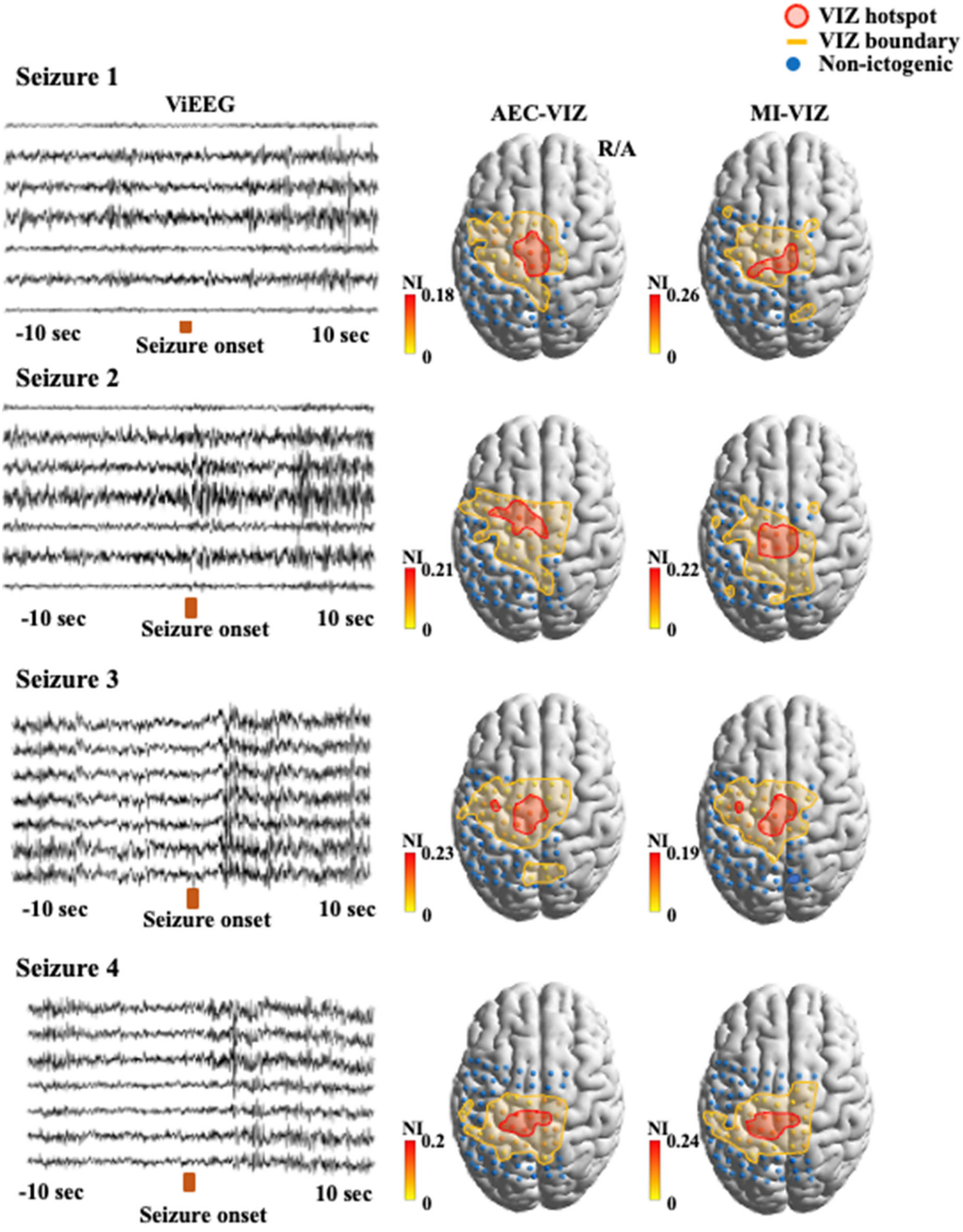

VIZ hotspot
VIZ boundary
Non-ictogenic
Seizure 1
ViEEG
AEC-VIZ
MI-VIZ
R/A
NI
0.18
0
0.26
-10 sec
Seizure onset
10 sec
Seizure 2
0.21
0.22
Seizure 3
0.23
0.19
Seizure 4
0.2
0.24

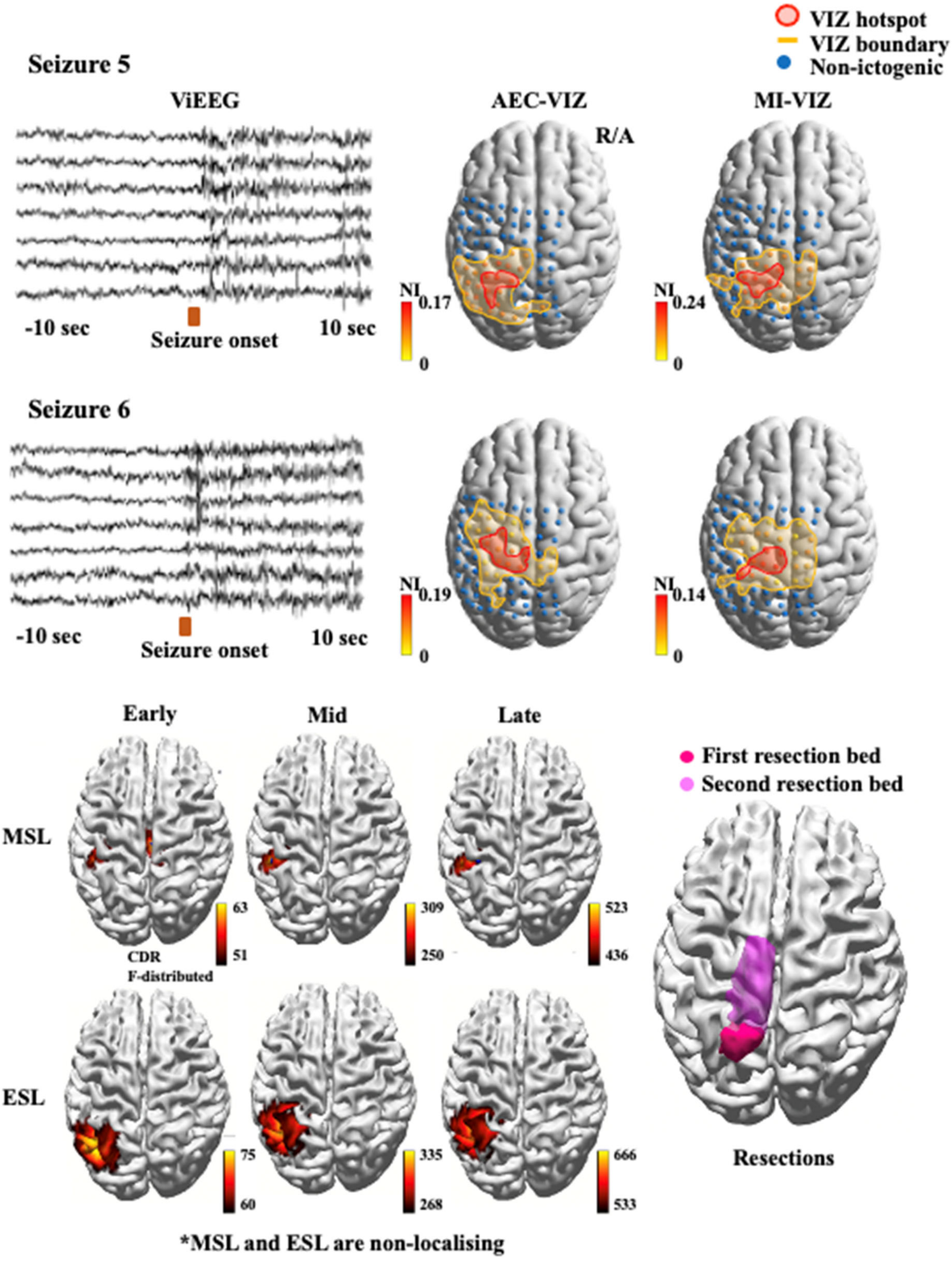

Supplementary Figure 7. Patient 3 had a normal MRI with over 100 seizure events per month before surgery and infrequent non-disabling seizures at 23 months post-surgery (Engel I).

MEG sLORETA ictal early source localisation suggested a focus at the left paracentral lobule. Guided by MSL solutions, ViEEGs were defined to extensively cover vertex, left parietal, and lateral temporal areas. Source signals of seven MEG captured seizure events are reconstructed while six seizures present distinct morphology of ictal spikes. Ictal discharges can be seen in the representative ViEEG channels with a spatial distribution that is similar across the seizure events. Both AEC-VIZ and MI-VIZ identified hotspots (nodes in red line and shade) encompassing the left paracentral lobule. This area is highlighted by five seizures (Seizure 1, Seizure 2, Seizure 3, Seizure 4, Seizure 6). AEC-VIZ derived from Seizure 5 spreads laterally while MI-VIZ extends medially. Therefore, both AEC-VIZ and MI-VIZ from MEG data better concord with the earliest solution given by MSL. This patient has achieved Engel I outcome with rare non-disabling seizures. Based on the surgical outcome, the MEG derived AEC-VIZ and MI-VIZ successfully captured the bulk of the likely EZ.

Abbreviations: MEG = magnetoencephalography, iEEG = intracranial electroencephalography , ViEEG =virtual intracranial electroencephalography , EZ = epileptogenic zone, SOZ = seizure onset zone, HDEEG =high density electroencephalography, VIZ = virtual ictogenic zone, MSL = MEG source localisation, ESL =HDEEG source localisation, AEC = amplitude envelope correlation, MI = mutual information, AEC-VIZ =virtual ictogenic zone using amplitude envelope correlation, MI-VIZ = virtual ictogenic zone using mutual information, NI = node ictogenicity.

Patient 4

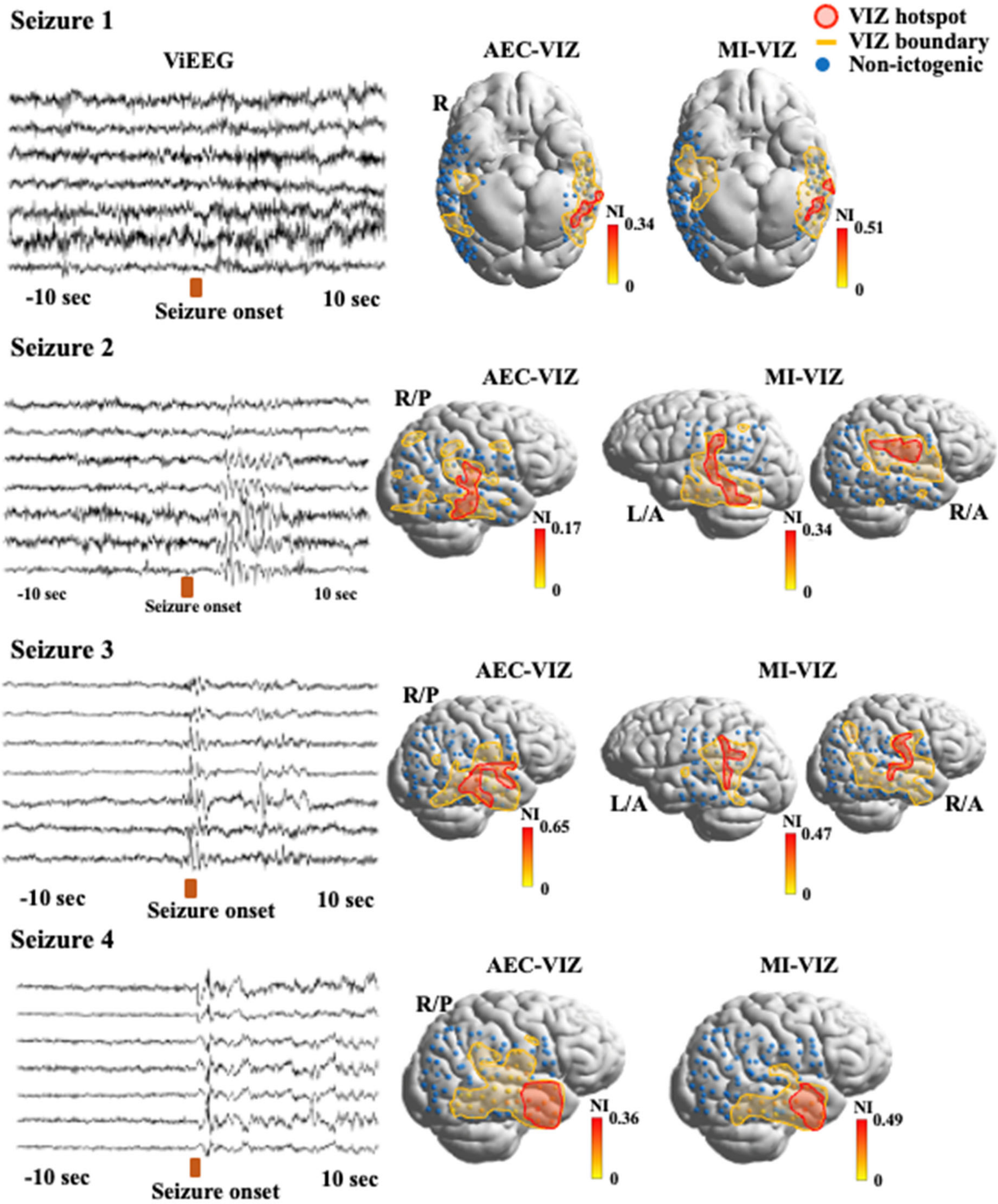

Seizure 1
ViEEG
AEC-VIZ
MI-VIZ
VIZ hotspot
VIZ boundary
Non-ictogenic
R
NI 0.34
0
NI 0.51
0
-10 sec
Seizure onset
10 sec
Seizure 2
AEC-VIZ
MI-VIZ
R/P
L/A
R/A
NI 0.17
0
NI 0.34
0
-10 sec
Seizure onset
10 sec
Seizure 3
AEC-VIZ
MI-VIZ
R/P
L/A
R/A
NI 0.65
0
NI 0.47
0
-10 sec
Seizure onset
10 sec
Seizure 4
AEC-VIZ
MI-VIZ
R/P
NI 0.36
0
NI 0.49
0
-10 sec
Seizure onset
10 sec

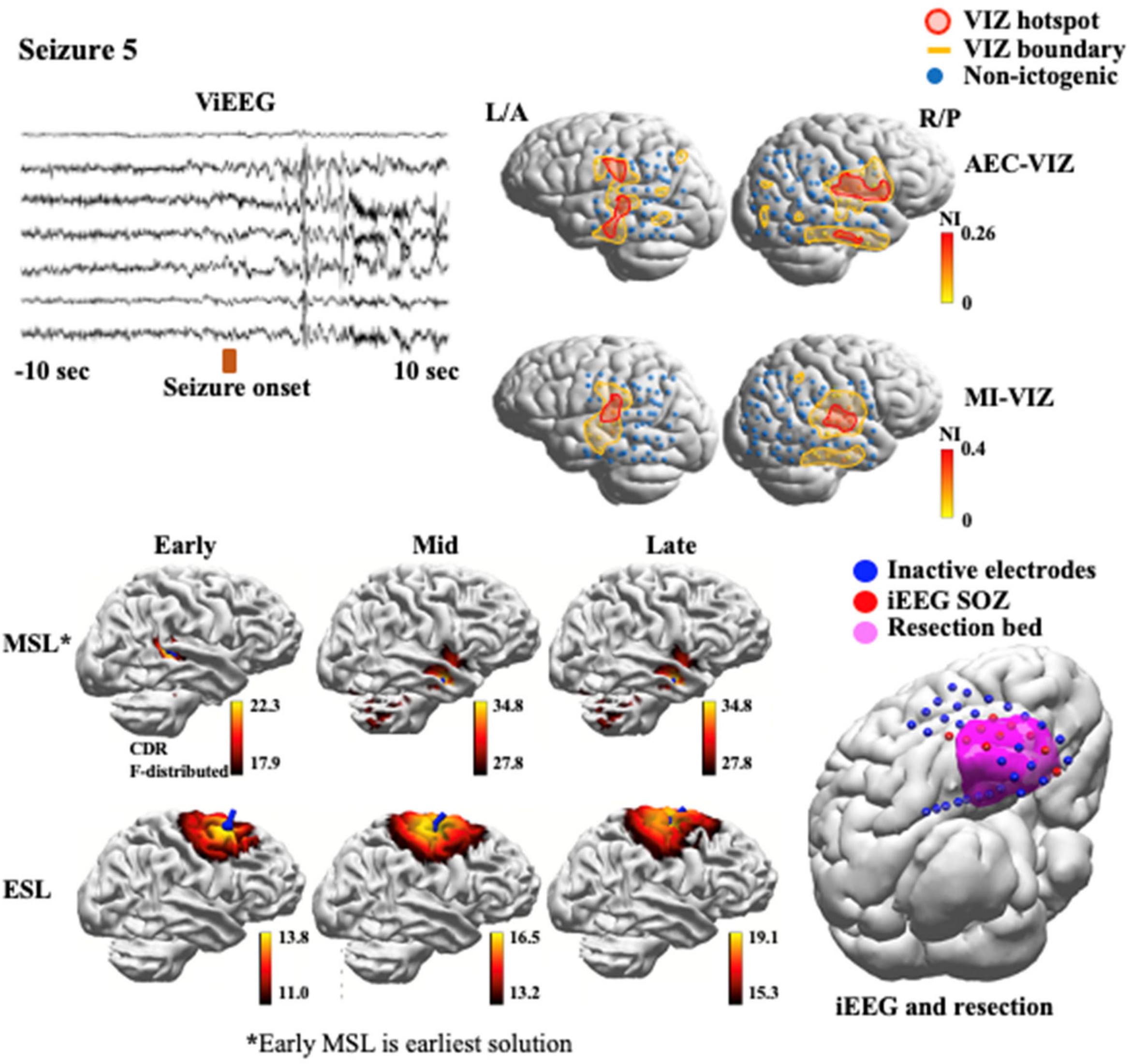

Supplementary Figure 8. Patient 4 had a normal MRI with over 20 seizure events per month before surgery. After surgery, the patient had infrequent disabling and non-disabling seizures at 21 months follow-up. MEG sLORETA ictal early source localisation suggested a focus at the posterior superior temporal gyrus. Guided by MSL solutions, ViEEGs were defined to extensively cover bilateral temporal, parietal, and occipital areas in a non-regularized pattern to enhance detection of VIZ beyond regular iEEG configurations. Source signals of eight MEG captured seizure events are reconstructed while five seizures present distinct morphology of ictal spikes. Ictal discharges can be seen in the representative ViEEG channels. AEC-VIZ and MI-VIZ identified different hotspots using Seizure 2, Seizure 3, and Seizure 5, while AEC-VIZ and MI-VIZ hotspots agree in Seizure 1 and Seizure 4. Variability of AEC-

VIZ and MI-VIZ boundaries is observed between seizures; multiple, bilateral regions are identified to be VIZ hotspots (high NI values). This patient has achieved Engel III outcome with fewer disabling seizures. Based on the suboptimal surgical outcome, the MEG derived AEC-VIZ and MI-VIZ may suggest epileptic foci outside the current resection.

Abbreviations: MEG = magnetoencephalography, iEEG = intracranial electroencephalography , ViEEG =virtual intracranial electroencephalography , EZ = epileptogenic zone, SOZ = seizure onset zone, HDEEG =high density electroencephalography, VIZ = virtual ictogenic zone, MSL = MEG source localisation, ESL =HDEEG source localisation, AEC = amplitude envelope correlation, MI = mutual information, AEC-VIZ =virtual ictogenic zone using amplitude envelope correlation, MI-VIZ = virtual ictogenic zone using mutual information, NI = node ictogenicity.

Patient 5

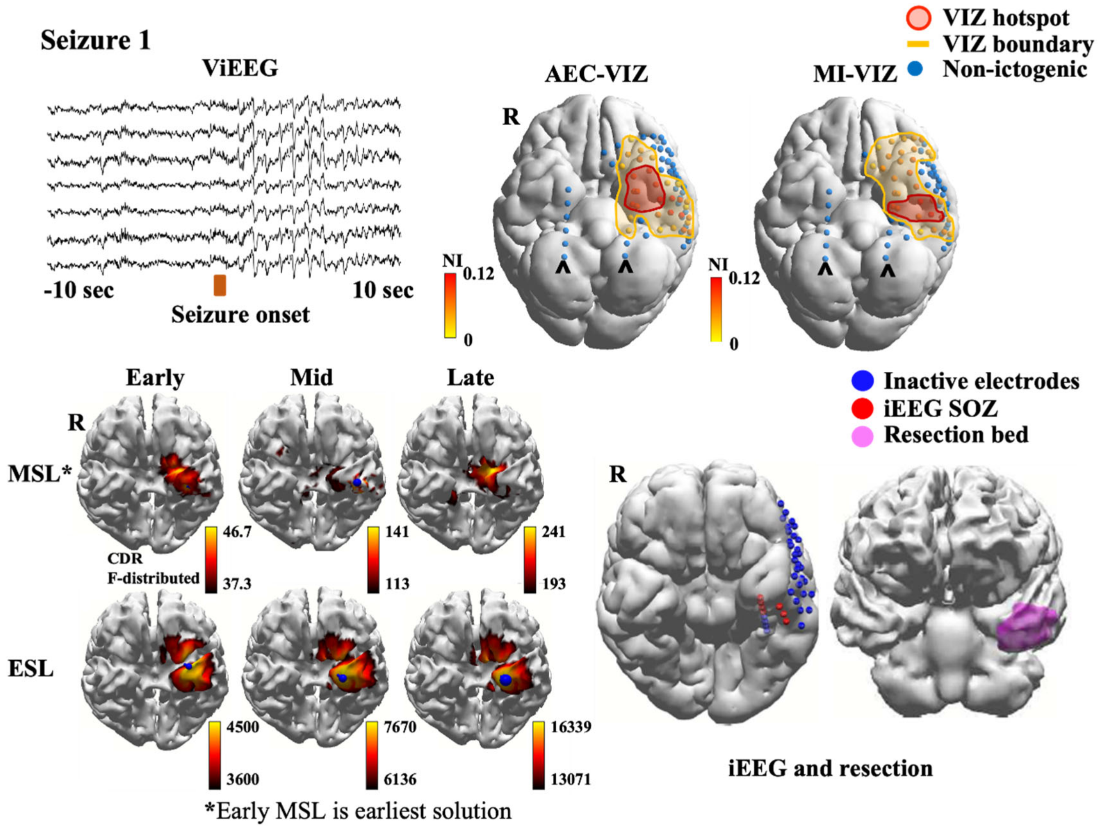

Supplementary Figure 9. Patient 5 had a normal MRI with over 20 seizures per month before surgery and is seizure free at 20 months follow-up. MEG sLORETA ictal source localisation suggested a focus at the mesial temporal region before propagation to the temporal pole. Guided by MSL solutions, ViEEGs were defined to extensively cover basal, inferior and lateral temporal areas. Two depth electrode-like ViEEGs were also defined to cover both hippocampi. Source signals of two MEG captured seizures are reconstructed while only the first seizure presents distinct morphology for ictal spikes. Ictal discharges can be seen in the representative ViEEG channels. Both AEC-VIZ and MI-VIZ identified hotspots (nodes in red line and shade) encompassing the left mesio-basal temporal region. AEC-VIZ and MI-VIZ hotspots concord with both MSL and ESL solutions. However, the extent of both AEC-VIZ and MI-VIZ are broader than resection margins and MSL solutions. This patient has achieved Engel I seizure-free outcome. Based on the seizure-free outcome, the MEG derived AEC-VIZ and MI-VIZ successfully captured the proposed EZ.

Abbreviations: MEG = magnetoencephalography, iEEG = intracranial electroencephalography , ViEEG =virtual intracranial electroencephalography , EZ = epileptogenic zone, SOZ = seizure onset zone, HDEEG =high density

electroencephalography, VIZ = virtual ictogenic zone, MSL = MEG source localisation, ESL =HDEEG source localisation, AEC = amplitude envelope correlation, MI = mutual information, AEC-VIZ =virtual ictogenic zone using amplitude envelope correlation, MI-VIZ = virtual ictogenic zone using mutual information, NI = node ictogenicity.

Patient 6

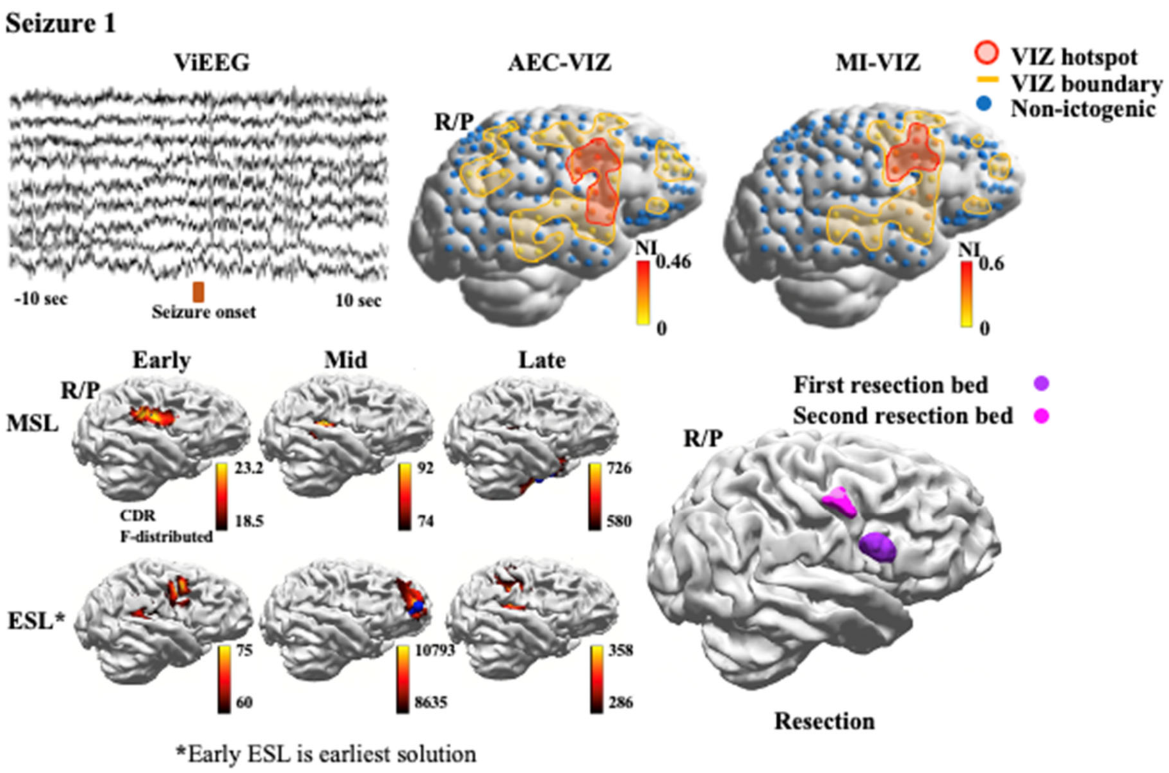

Supplementary Figure 10. Patient 6 had a normal MRI with over 100 seizures per month before surgery, which led to an Engel I outcome. MEG and EEG sLORETA ictal early source localisation suggested a focus in the region of the right central sulcus and pre-motor cortex. Guided by MSL solutions, ViEEGs were defined to extensively cover lateral parietal, temporal, and frontal areas including the frontal pole. Source signals of MEG captured continuous spikes are reconstructed. Continuous spikes can be seen in the representative ViEEG channels. AEC-VIZ and MI-VIZ identified hotspots (nodes in red line and shade) encompassing a focus at the right pre-motor cortex. Note the overlap with the early ESL solution and the surgical resection and the lack of overlap with the MSL solutions. Compared to the AEC-VIZ hotspot, the MI-VIZ hotspot is, however, more localised to the early ESL solution and to the successful second resection (while the AEC-VIZ also includes the failed first resection). Nonetheless, AEC-VIZ and MI-VIZ from MEG data better concords with the earliest solution given by the EEG rather than the corresponding MEG sLORETA solution. This patient has achieved Engel I seizure-free outcome according to the latest review (over two years since surgery that showed a cortical dysplasia). Based on the surgical outcome, the

MEG derived AEC-VIZ and MI-VIZ successfully captured the putative EZ, while the MEG derived sLORETA solution did not.

Abbreviations: MEG = magnetoencephalography, iEEG = intracranial electroencephalography , ViEEG =virtual intracranial electroencephalography , EZ = epileptogenic zone, SOZ = seizure onset zone, HDEEG =high density electroencephalography, VIZ = virtual ictogenic zone, MSL = MEG source localisation, ESL =HDEEG source localisation, AEC = amplitude envelope correlation, MI = mutual information, AEC-VIZ =virtual ictogenic zone using amplitude envelope correlation, MI-VIZ = virtual ictogenic zone using mutual information, NI = node ictogenicity.

Patient 7

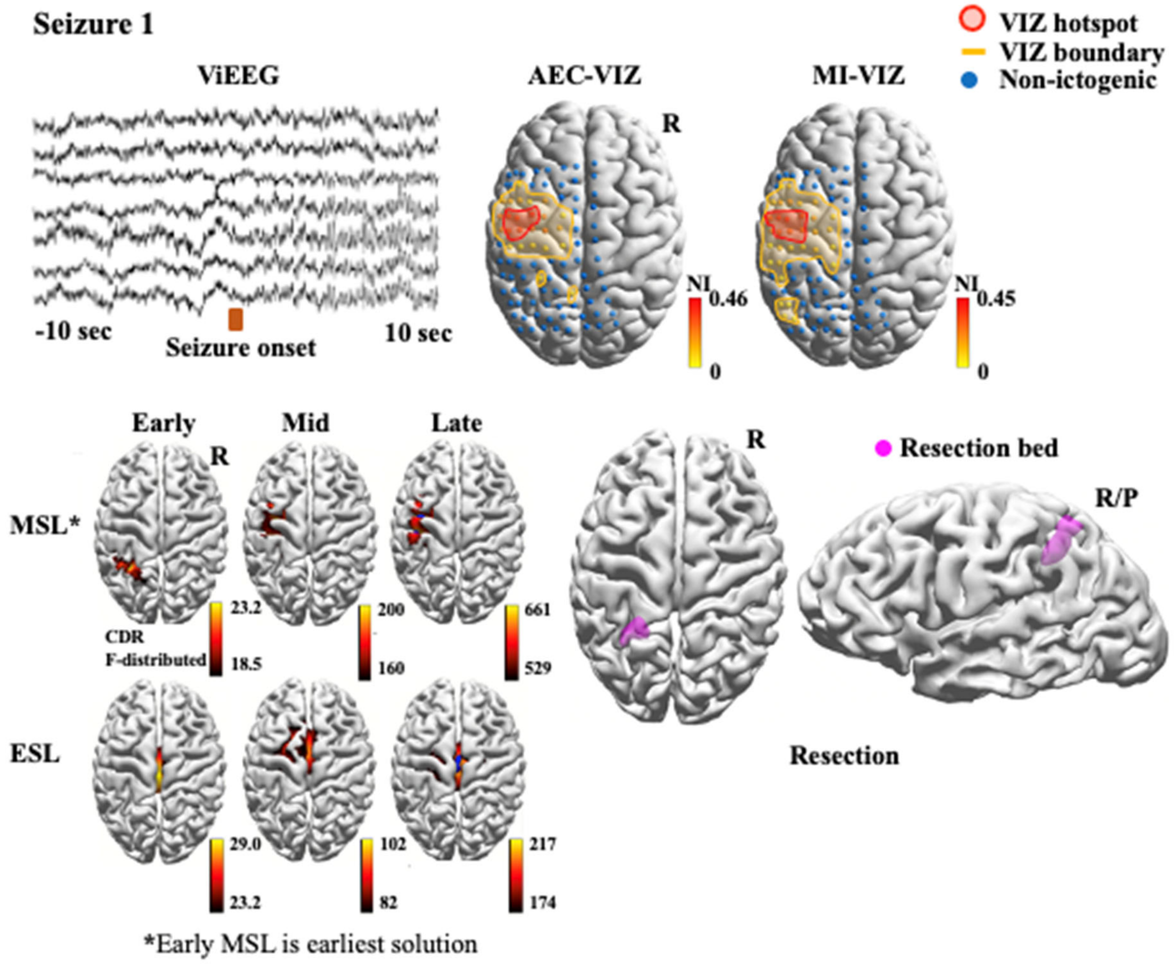

Supplementary Figure 11. Patient 7 had a normal MRI with over 100 seizures per month before surgery and is seizure-free at 20 months follow-up. EEG and MEG sLORETA source localisation of ictal discharges was non-localising while MEG sLORETA early source localisation of interictal discharges suggested a focus at the junction of post-central sulcus

and superior parietal lobule (ESL and MSL solutions using interictal spikes shown). Guided by MSL solutions using interictal spikes, ViEEGs were defined to extensively cover frontal, parietal and anterior occipital areas. Source signals of a MEG captured seizure is reconstructed. Ictal discharges can be seen in the representative ViEEG channels. Both AEC and MI-VIZ identified hotspots (nodes in red line and shade) encompassing a localised area of the left central sulcus extending laterally. AEC-VIZ and MI-VIZ do not overlap the early-MSL (interictal) nor the surgical resection but better concords with mid-MSL (interictal) and late-MSL (interictal).This patient has achieved Engel I seizure-free outcome (histology was cortical dysplasia). Based on the seizure free outcome, the MEG derived AEC and MI-VIZ did not capture the likely EZ.

Abbreviations: MEG = magnetoencephalography, iEEG = intracranial electroencephalography , ViEEG =virtual intracranial electroencephalography , EZ = epileptogenic zone, SOZ = seizure onset zone, HDEEG =high density electroencephalography, VIZ = virtual ictogenic zone, MSL = MEG source localisation, ESL =HDEEG source localisation, AEC = amplitude envelope correlation, MI = mutual information, AEC-VIZ =virtual ictogenic zone using amplitude envelope correlation, MI-VIZ = virtual ictogenic zone using mutual information, NI = node ictogenicity.

Patient 8

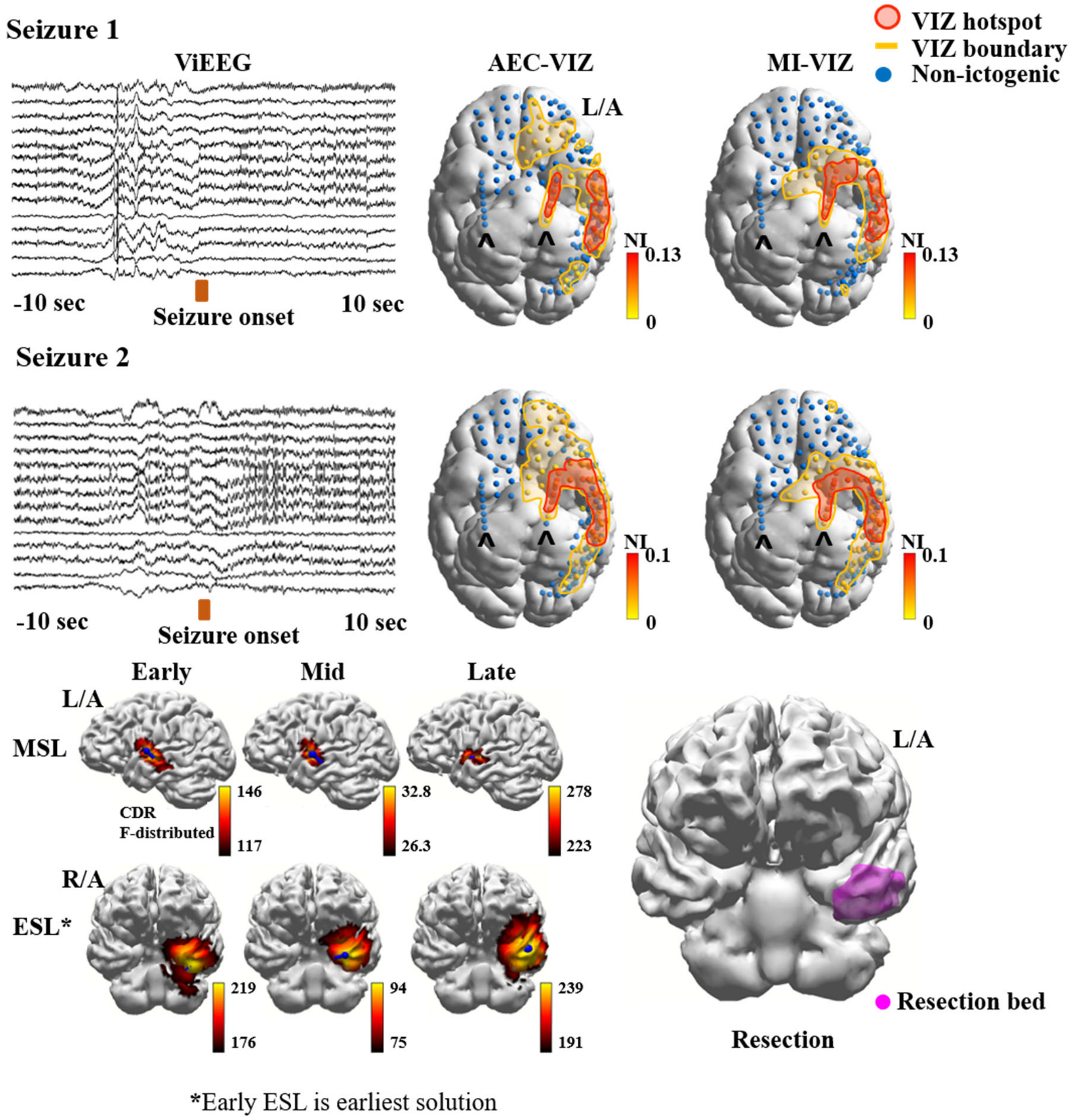

Supplementary Figure 12. Patient 8 had a normal MRI with over 20 seizures per month before surgery and is seizure free at 22 months follow-up. MEG sLORETA ictal early source localisation suggested a focus at the left superior temporal gyrus. Based on this, ViEEGs were defined to extensively cover left lateral temporal, basal temporal, temporal pole, orbitofrontal, and frontal pole surfaces. Source signals of two MEG captured seizure events are reconstructed. Ictal discharges can be seen in the representative ViEEG channels with a spatial distribution that is similar across the two seizure events. Both AEC-VIZ and MI-VIZ

identified hotspot (nodes in red line and shade) encompassing the left basal and mesial temporal structures including anterior hippocampus and temporal pole. Note the overlap with the ESL solutions and the surgical resection and the lack of overlap with the MSL solutions. Hence, AEC-VIZ and MI-VIZ from MEG data better concords with the earliest solution given by the EEG rather than the corresponding MEG sLORETA solution. This patient has achieved Engel I seizure-free outcome according to the latest review (cortical dysplasia on histology). Based on the seizure free outcome, the MEG derived AEC-VIZ and MI-VIZ successfully captured the putative EZ while the MEG derived sLORETA solution, that sat well outside the resection bed, did not. (ViEEG hippocampal depth electrodes are denoted by hat symbols to distinguish them from the ViEEG grid electrodes).

Abbreviations: MEG = magnetoencephalography, iEEG = intracranial electroencephalography , ViEEG =virtual intracranial electroencephalography , EZ = epileptogenic zone, SOZ = seizure onset zone, HDEEG =high density electroencephalography, VIZ = virtual ictogenic zone, MSL = MEG source localisation, ESL =HDEEG source localisation, AEC = amplitude envelope correlation, MI = mutual information, AEC-VIZ =virtual ictogenic zone using amplitude envelope correlation, MI-VIZ = virtual ictogenic zone using mutual information, NI = node ictogenicity.

Patient 9

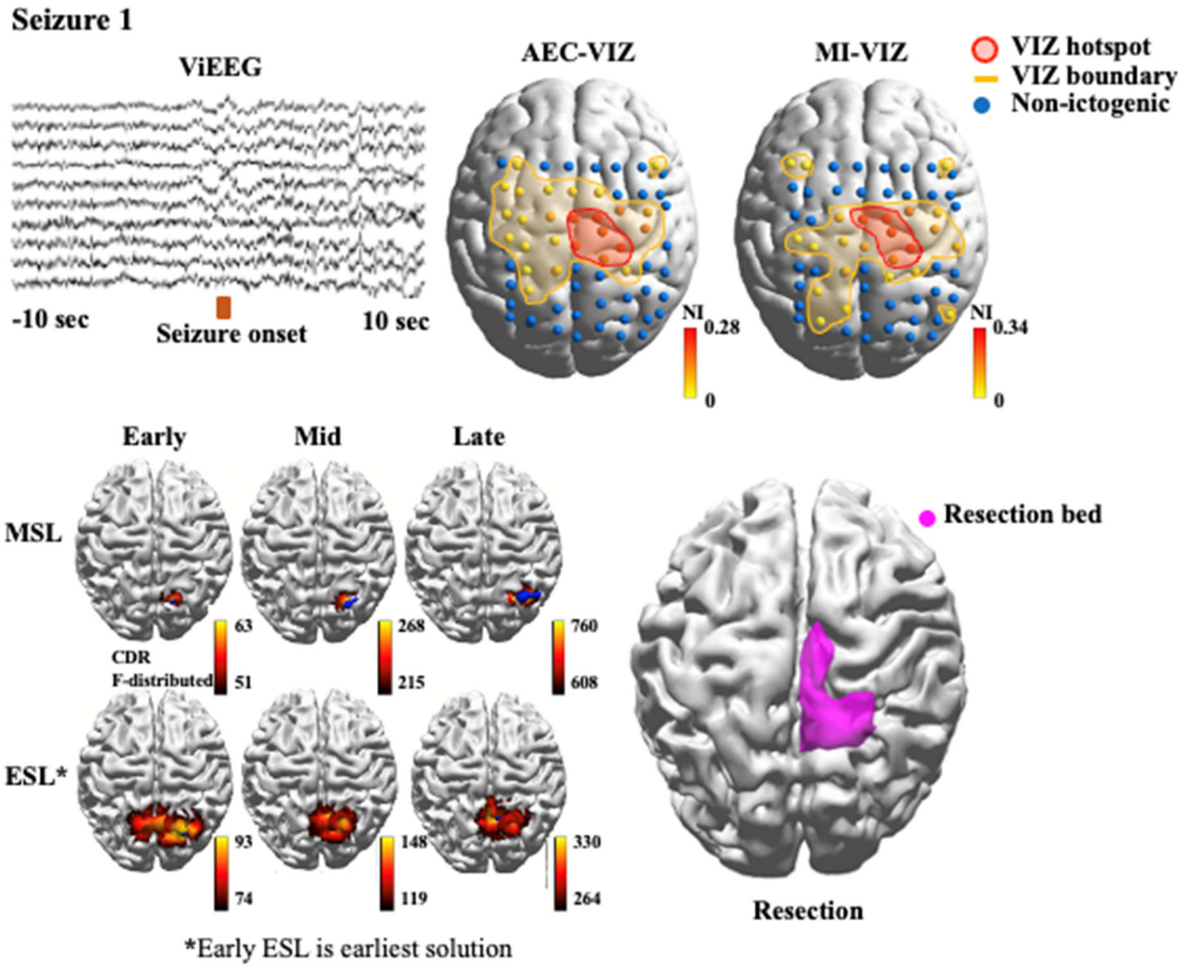

Supplementary Figure 13. Patient 9 had a normal MRI with over 100 seizures per month (motor left leg) before surgery. After surgery, the patient was seizure free for 6 months but then developed new left arm motor events. MEG sLORETA ictal early source localisation suggested a focus at the right posterior paracentral lobule and precuneus. Guided by MSL solutions, ViEEGs were defined to extensively cover the vertex, biparietal and posterior bifrontal areas. Source signals of two MEG captured seizures are reconstructed while only the first seizure presents a distinct morphology for ictal spikes. Ictal discharges can be seen in the representative ViEEG channels. Both AEC and MI-VIZ identified hotspots (nodes in red line and shade) encompassing the right paracentral lobule which does not concord with MSL or ESL solutions. This patient has achieved Engel II outcome with rare disabling seizures. The MEG derived AEC-VIZ and MI-VIZ overlap the surgical resection (that showed cortical dysplasia) but the return of seizures suggests that the EZ was not sufficiently removed.

Abbreviations: MEG = magnetoencephalography, iEEG = intracranial electroencephalography , ViEEG =virtual intracranial electroencephalography , EZ = epileptogenic zone, SOZ = seizure onset zone, HDEEG =high density electroencephalography, VIZ = virtual ictogenic zone, MSL = MEG source localisation, ESL =HDEEG source localisation, AEC = amplitude envelope correlation, MI = mutual information, AEC-VIZ =virtual ictogenic zone using amplitude envelope correlation, MI-VIZ = virtual ictogenic zone using mutual information, NI = node ictogenicity.

Patient 10

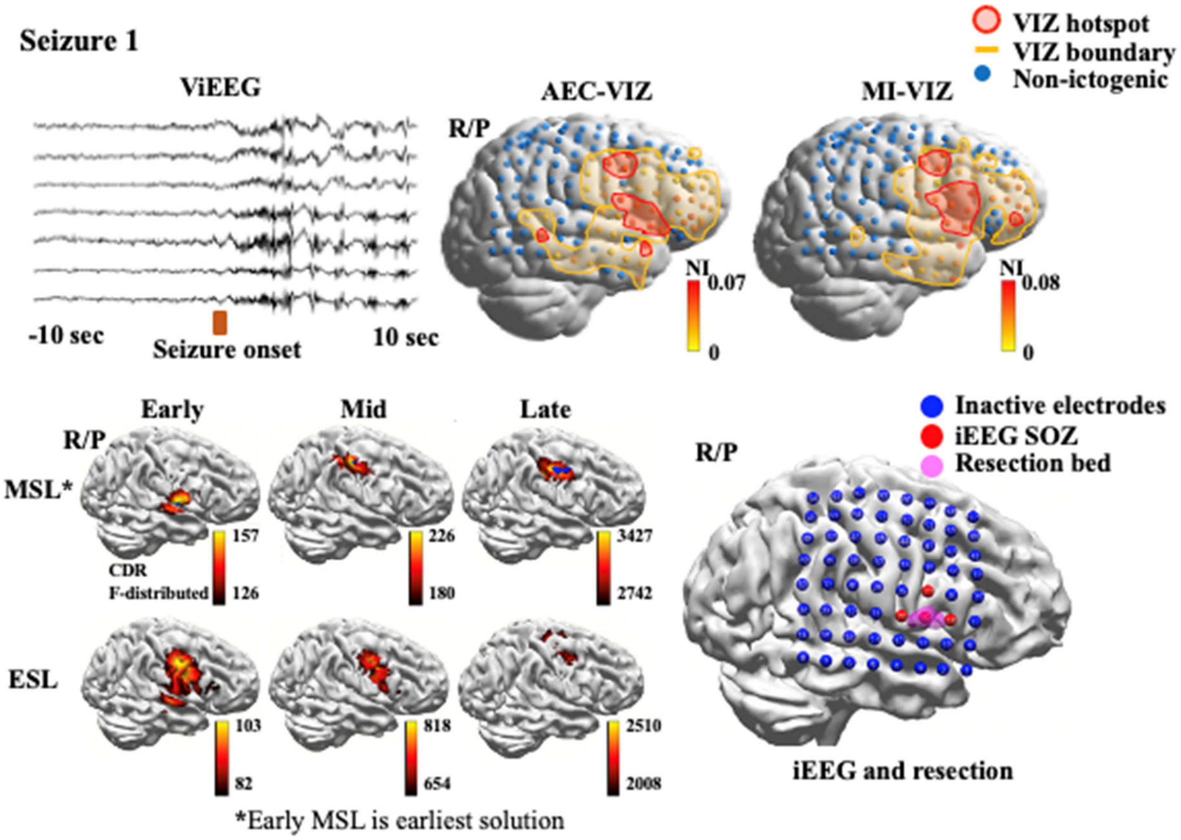

Supplementary Figure 14. Patient 10 had an extensive cortical dysplasia of the right fronto-temporo-parietal area with over 100 seizures per month before surgery, which was successful in stopping her disabling events. MEG sLORETA ictal early source localisation suggested a focus at the base of the pre-central gyrus. Guided by MSL solutions and by the extent of the dysplasia, ViEEGs were defined to extensively cover much of the right hemisphere. Source signals of MEG captured continuous spikes that are reconstructed and shown in the representative ViEEG channels. Both AEC-VIZ and MI-VIZ identified hotspots (nodes in red line and shade) containing two isolated areas (one inferior and the other superior) at the lateral

frontal convexity where the inferior hotspot better concords with the resection margin, iEEG SOZ and the earliest solution given by MEG while the superior hotspot overlaps with the late-MSL and late-ESL. This patient has achieved Engel I outcome with non-disabling seizures. Based on surgical outcome, the MEG derived AEC-VIZ and MI-VIZ successfully captured the surgical resection and may represent the wider extent of the suspected EZ.

Abbreviations: MEG = magnetoencephalography, iEEG = intracranial electroencephalography , ViEEG =virtual intracranial electroencephalography , EZ = epileptogenic zone, SOZ = seizure onset zone, HDEEG =high density electroencephalography, VIZ = virtual ictogenic zone, MSL = MEG source localisation, ESL =HDEEG source localisation, AEC = amplitude envelope correlation, MI = mutual information, AEC-VIZ =virtual ictogenic zone using amplitude envelope correlation, MI-VIZ = virtual ictogenic zone using mutual information, NI = node ictogenicity.

Patient 11

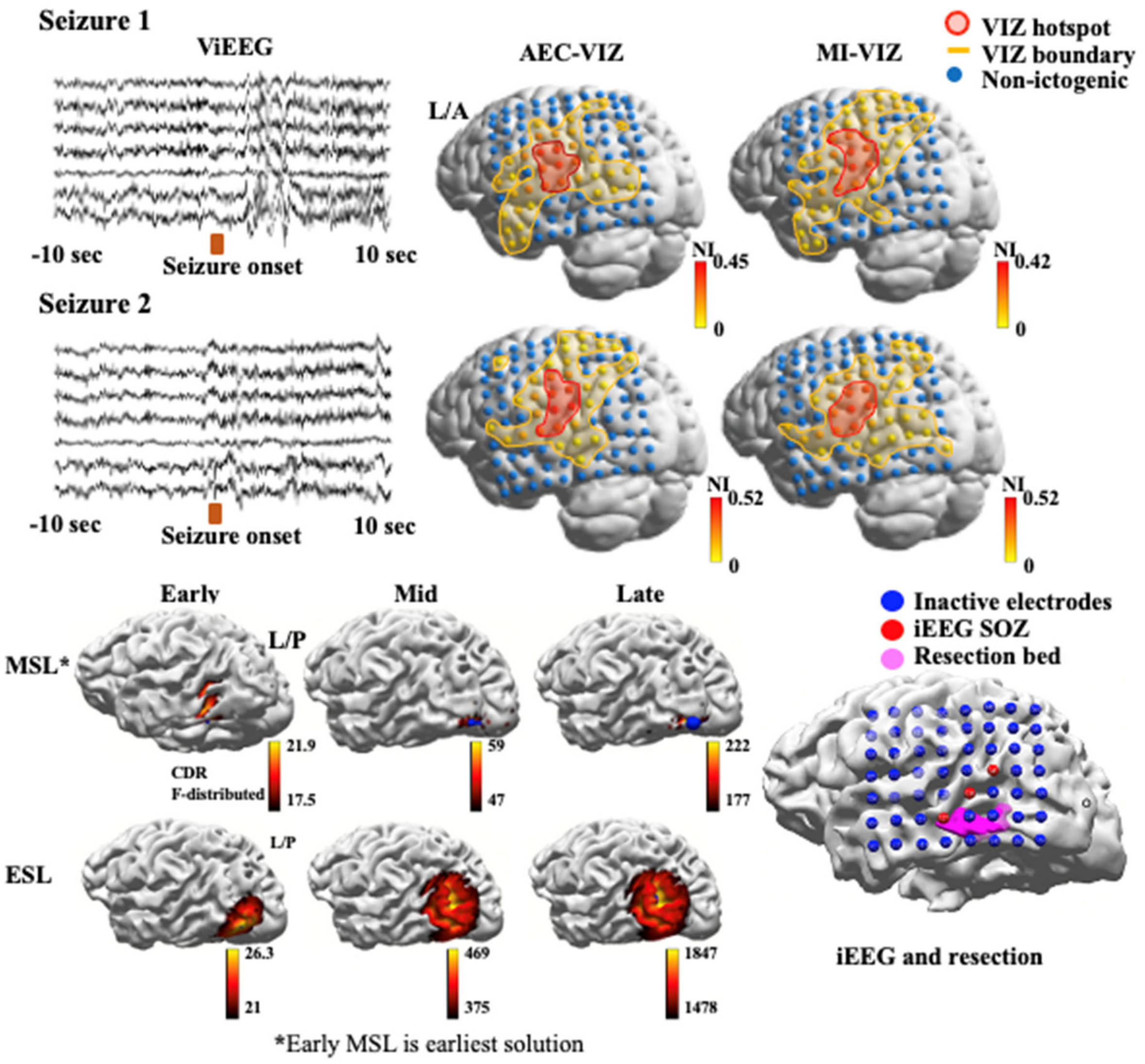

Supplementary Figure 15. Patient 11 had an extensive lesion at the left temporo-parieto-occipital (TPO) area on MRI with frequent disabling seizures (average 15 seizures per month) before surgery. Resection only gave an Engel III outcome. MEG sLORETA ictal early source localisation suggested a focus at the TPO junction. Guided by MSL solutions, ViEEGs were defined to extensively cover TPO junction and posterior frontal, superior parietal and lateral temporal areas. Source signals of two MEG captured seizure events are reconstructed. Ictal discharges can be seen in the representative ViEEG channels. Both AEC-VIZ and MI-VIZ identified hotspots (nodes in red line and shade) encompassing the left parieto-temporal convexity, with their boundaries including the TPO junction. AEC-VIZ and MI-VIZ from MEG data better concords with the earliest solution given by the MEG. This patient has achieved Engel III outcome with fewer disabling seizures. Based on surgical outcome, the MEG derived AEC-VIZ and MI-VIZ may represent the wider extent of the suspected EZ. Indeed, the iEEG SOZ was concordant with the VIZ solutions and extended beyond the limited resection zone. The resection area was limited by adjacent eloquent visual tracts.

Abbreviations: MEG = magnetoencephalography, iEEG = intracranial electroencephalography , ViEEG =virtual intracranial electroencephalography , EZ = epileptogenic zone, SOZ = seizure onset zone, HDEEG =high density electroencephalography, VIZ = virtual ictogenic zone, MSL = MEG source localisation, ESL =HDEEG source localisation, AEC = amplitude envelope correlation, MI = mutual information, AEC-VIZ =virtual ictogenic zone using amplitude envelope correlation, MI-VIZ = virtual ictogenic zone using mutual information, NI = node ictogenicity.

Patient 12

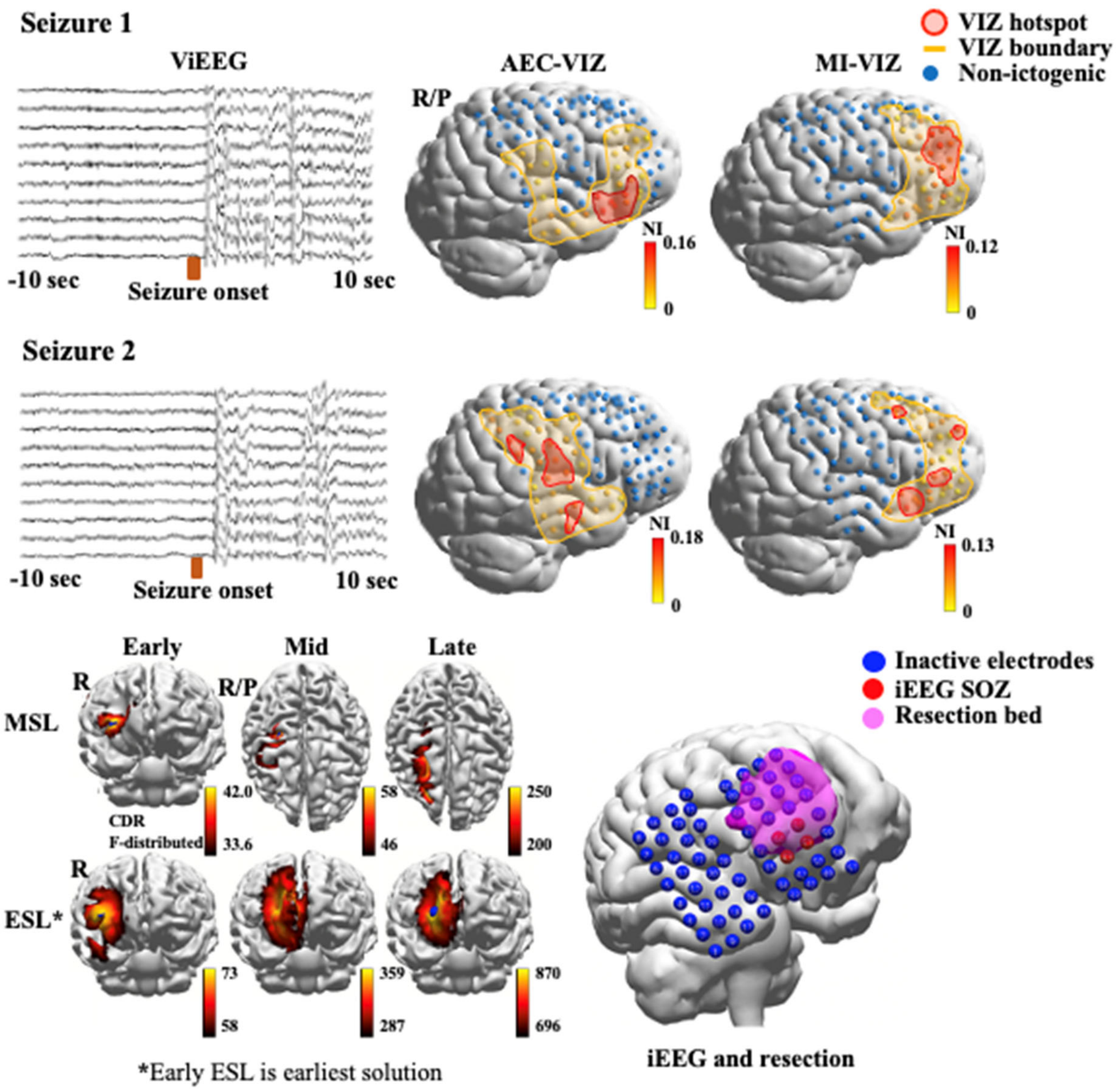

Supplementary Figure 16. Patient 12 had a large right frontal gliotic lesion with over 4 disabling seizures per month before resection which led to complete seizure freedom at 24 months. MEG sLORETA ictal early source localisation suggested a focus at right inferior frontal gyrus. Guided by MSL solutions, ViEEGs were defined to extensively cover right frontal, fronto-parietal and superior temporal areas. Source signals of two MEG captured seizure events are reconstructed. Ictal discharges can be seen in the representative ViEEG channels. Variability of hotspot and boundary results for AEC-VIZ and MI-VIZ is present across both seizures. MI-VIZ boundary from Seizure 1 and Seizure 2 fully contains the resection margin and the earliest solution given by the EEG rather than the MEG while MI-

VIZ hotspot from Seizure 1 better predicts the putative EZ, iEEG SOZ and early-ESL (the earliest solution in this case). AEC-VIZ is discordant with the resection margin, iEEG SOZ and source localisation solutions. This patient has achieved Engel I seizure-free outcome. Based on the seizure-free outcome, the MEG derived MI-VIZ successfully captured the likely EZ.

Abbreviations: MEG = magnetoencephalography, iEEG = intracranial electroencephalography , ViEEG =virtual intracranial electroencephalography , EZ = epileptogenic zone, SOZ = seizure onset zone, HDEEG =high density electroencephalography, VIZ = virtual ictogenic zone, MSL = MEG source localisation, ESL =HDEEG source localisation, AEC = amplitude envelope correlation, MI = mutual information, AEC-VIZ =virtual ictogenic zone using amplitude envelope correlation, MI-VIZ = virtual ictogenic zone using mutual information, NI = node ictogenicity.

ViEEG and iEEG Waveform Comparison

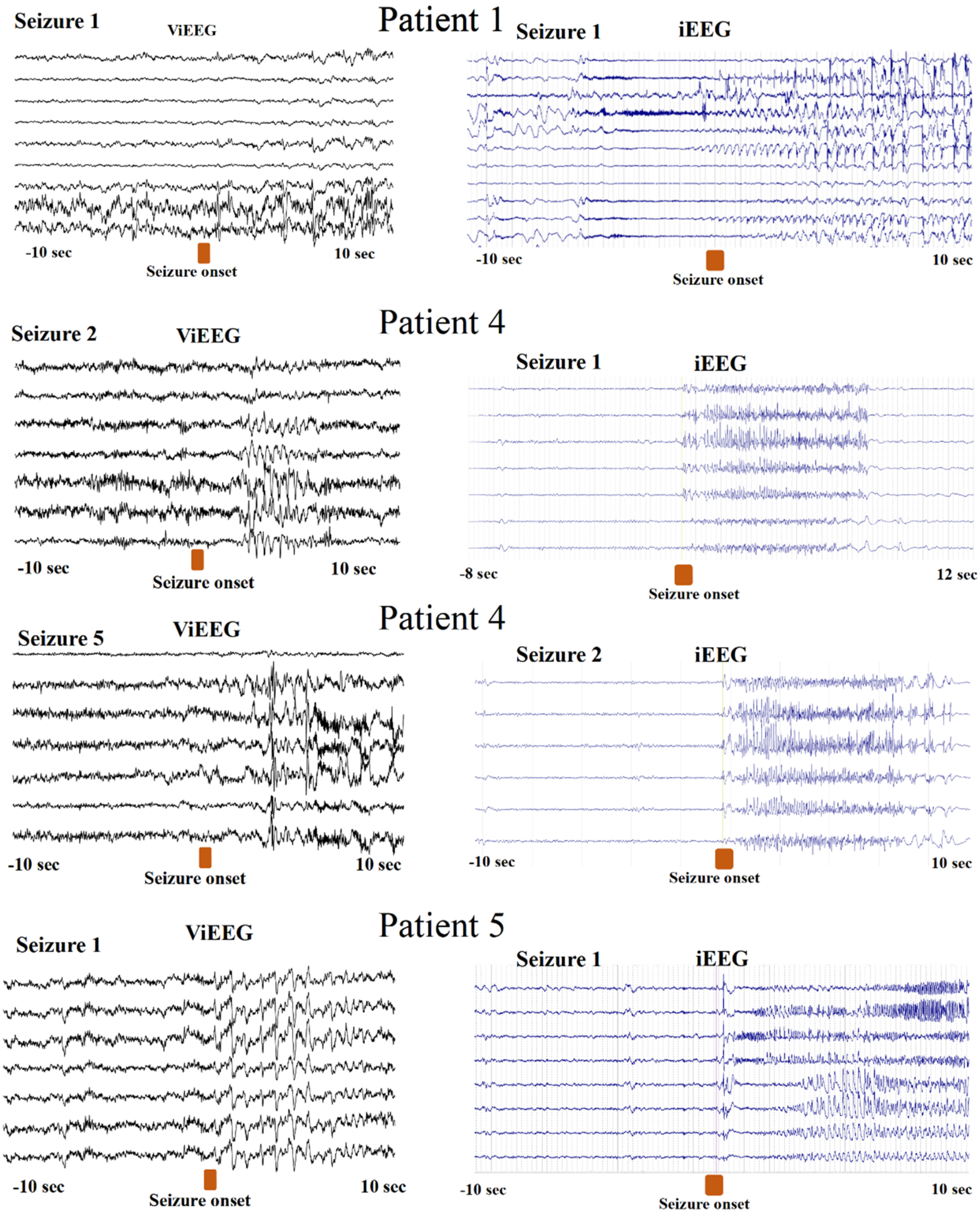

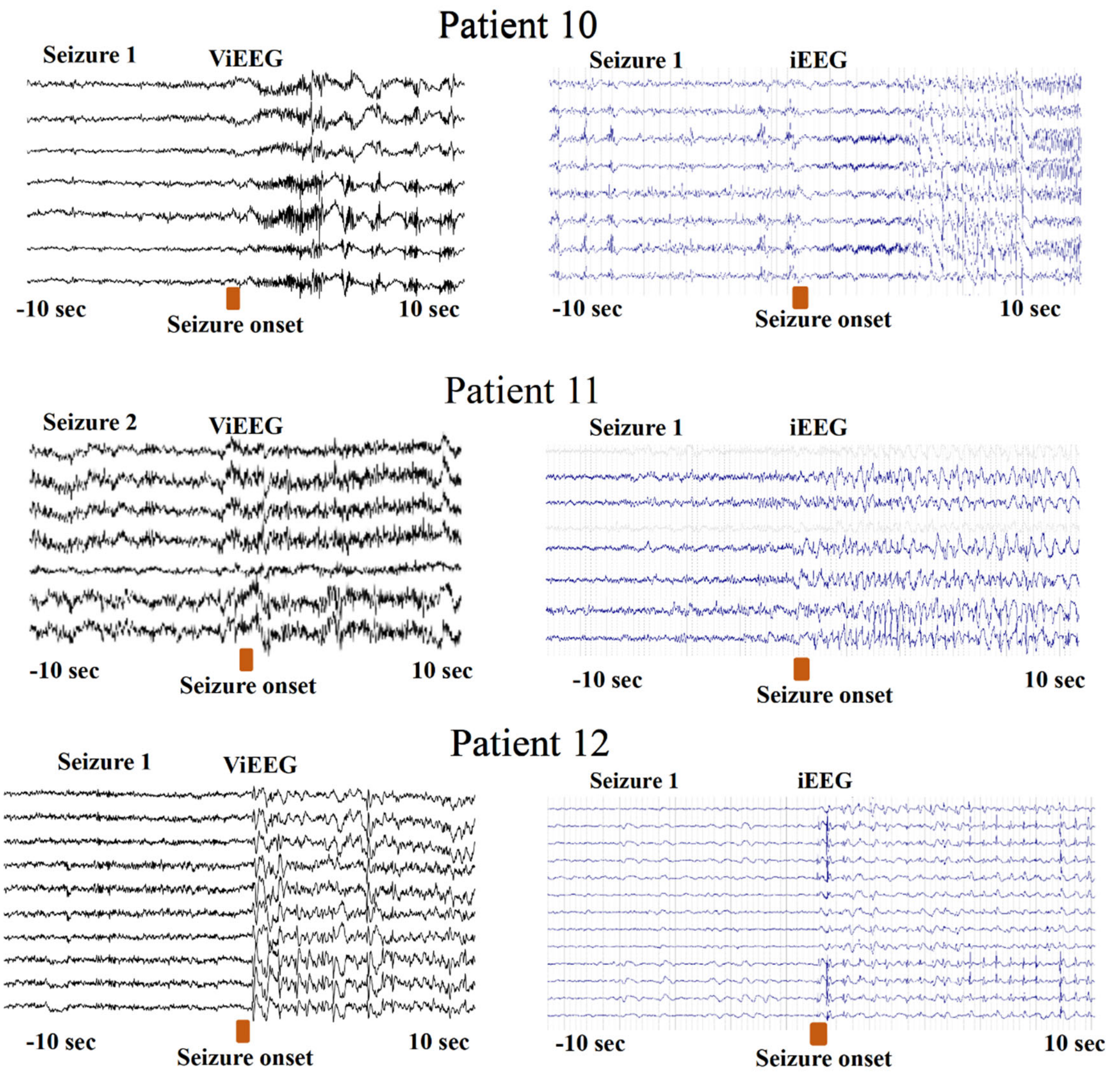

Supplementary Figure 17. Comparison of ictal ViEEG waveforms (left panels) against ictal iEEG waveforms (right panels) for patients whose iEEG data were available to us (Patients 1, 4, 5, 10, 11, 12). Ictal ViEEG and IEEG waveforms were plotted using 10 seconds before and after seizure onset for waveform morphology comparisons. ViEEG and iEEG samples were not acquired synchronously as MEG recordings were performed prior to iEEG implantation. Nonetheless, clinical features (semiology) of seizures were very similar for MEG and iEEG acquisitions.

Abbreviations: MEG = magnetoencephalography, iEEG = intracranial electroencephalography , ViEEG =virtual intracranial electroencephalography

Simulation Experiments

We used the Theta model to generate time series for establishing the validity of combining the beamforming technique and dynamical models to identify ictogenic nodes in a network setting. We note here that a full study will be required to fully understand the circumstances where ictal MEG signals are reconstructed using the beamforming technique; however, we establish that under some simple circumstances, we can establish these nodes.

Methods

Step 1) Use the theta model to generate 64 time series with 4 ictogenic nodes.

- θ_i^s , the distance from the SNIC (or steady state of θ_i), is set to -1.2 (the default used in the whole study) for ictogenic nodes. This value is lowered to -3 for other nodes. Therefore, non-seizure nodes are less likely to transition to the seizure state.
- The adjacency matrix CC_{ij} has strong connections ($CC_{ij} = 1$) between the ictogenic nodes and no connections otherwise. This coordinates the activity of the ictogenic nodes during the seizure state.

Step 2) Run the forward and inverse modelling on these time series (90 seconds included at 1000 Hz) to create transformed ("beamformed") time series.

- First, 64 time-series generated by the Theta model were assigned in the order of G01 to G64 (as shown in Supplementary Fig. 18) to the brain source space defined by the ViEEG grid (size of 8 by 8, spacing 10mm).
- Next, a realistic head model (inner skull surface, single layer) was constructed using the same Boundary Element Method (BEM) that has been used in the main manuscript. The head model was then used to generate the forward model to project source signals (64 time-series assigned to ViEEG grid locations) to MEG sensor signals (in our simulation, 306 MEG sensors).
- Then 64 time-series assigned to source space (defined by the ViEEG grid, Supplementary Fig. 18, Supplementary Fig. 19) are then projected to MEG sensor signals using the forward model. Additional multi-variate Gaussian noise is added to MEG sensor signals after the forward modelling. (Amplitudes of Gaussian noise were scaled relative to time-series generated by the Theta model based on SNR defined in the simulation; mean is set to zero and variance is defined by the covariance matrix derived from resting-state MEG signals acquired from a healthy subject).

- Last, the beamformers were constructed using the same Linear Constrained Minimum Variance (LCMV) technique (same parameters were applied as in the main manuscript) and were used to reconstruct the source signals at ViEEG grid locations (defined in Supplementary Fig. 18). Reconstructed ViEEG signals were then used for network analysis and dynamical modelling.

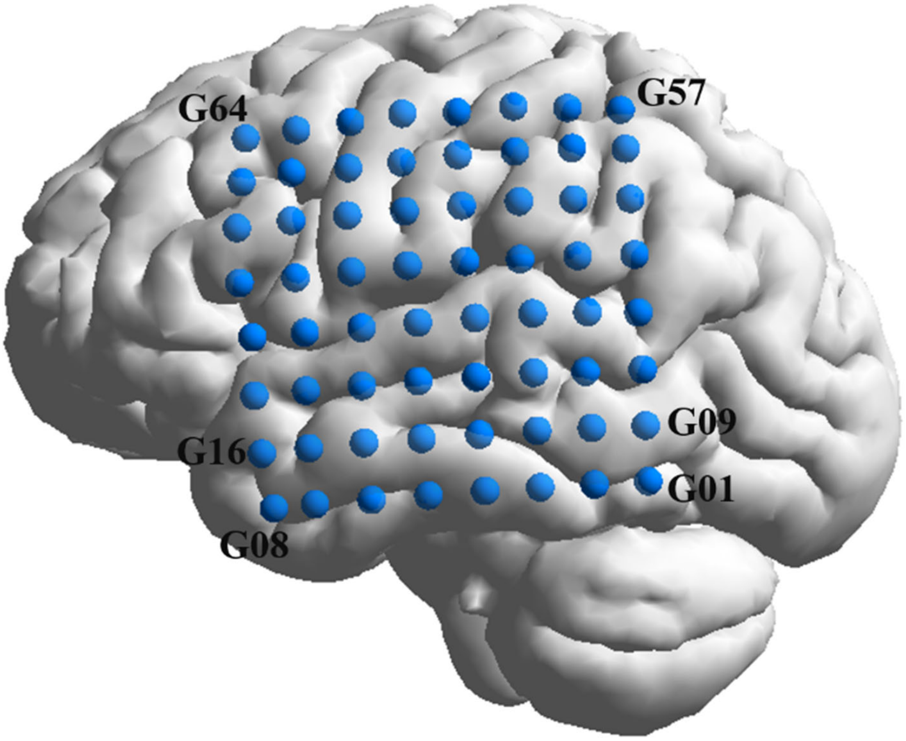

Supplementary Figure 18: ViEEG grid with size of 8-by-8 (64 nodes/time-series in total) placed in left temporal-parietal regions. Labels are assigned in the same fashion that G01 starts from posterior and inferior aspects.

Step 3) Use Mutual Information to identify functional connectivity networks for both the original time series and for the transformed time series (Supplementary Fig. 20).

Step 4) Identify the NI^i for each node and determine if large values of NI are the ictogenic nodes. We can use the original time series as the ground truth. (Supplementary Fig. 21)

Step 5) Repeat this procedure for 3 locations (central, upper left, lower right in the ViEEG grid, Supplementary Fig. 21, Supplementary Fig. 22, Supplementary Fig. 23).

Abbreviations: ViEEG =virtual intracranial electroencephalography

The code is available in the repository under the directory supplemental_materials_ground_truth.

Simulation Results

Example time series

Central example: nodes 28, 29, 36, 37.

Time units are arbitrary but can be thought of as seconds sampled at 1000 Hz for the beamformer. Plotted time series are standardised (each time series is mean-subtracted and divided by standard deviation)

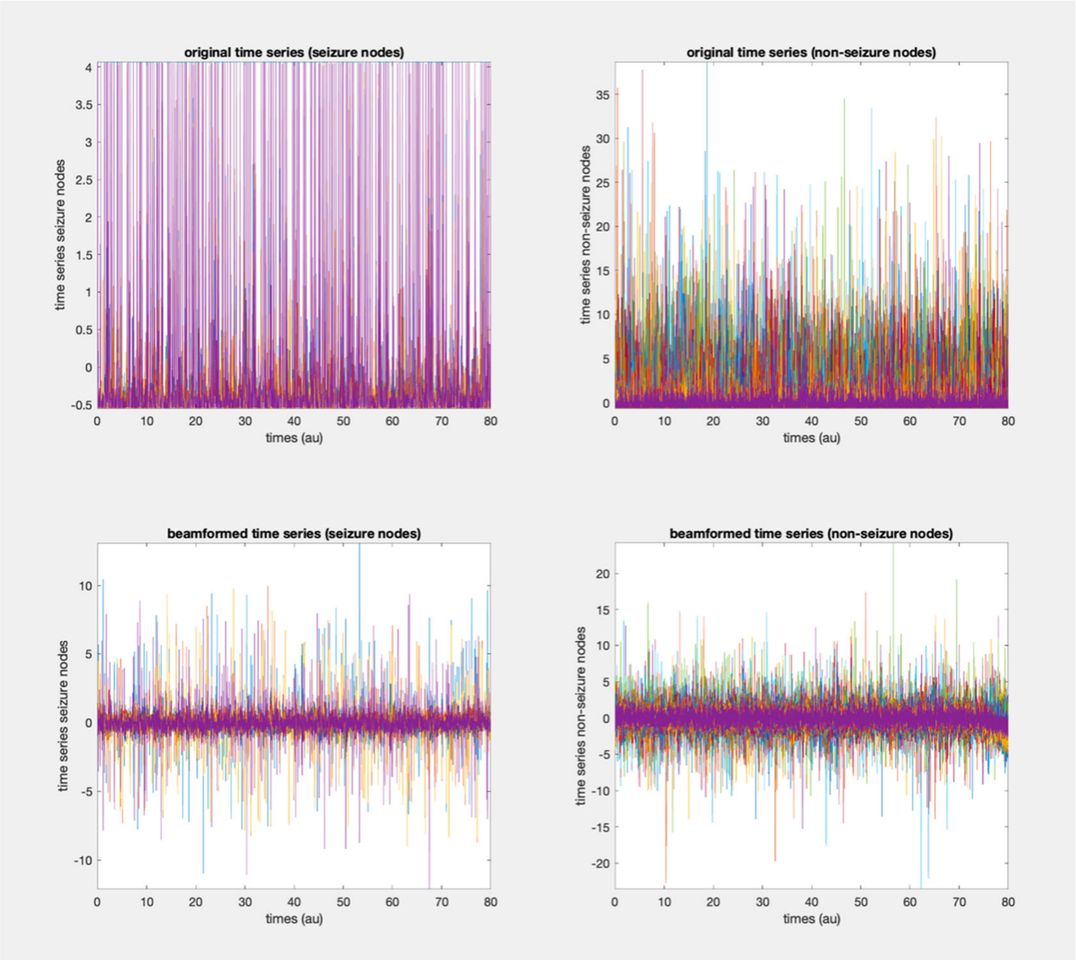

Supplementary Figure 19: Seizure and non-seizure nodes for original and beamformed time-series.

Functional Connectivity (and Structural Connectivity)

The structural connectivity refers to the connections between the 4 ictogenic nodes. The original functional connectivity (FC) closely matches the structural connectivity (below). We identify NI^i to assess if the ictogenic nodes can be found using the source-space FC matrix.

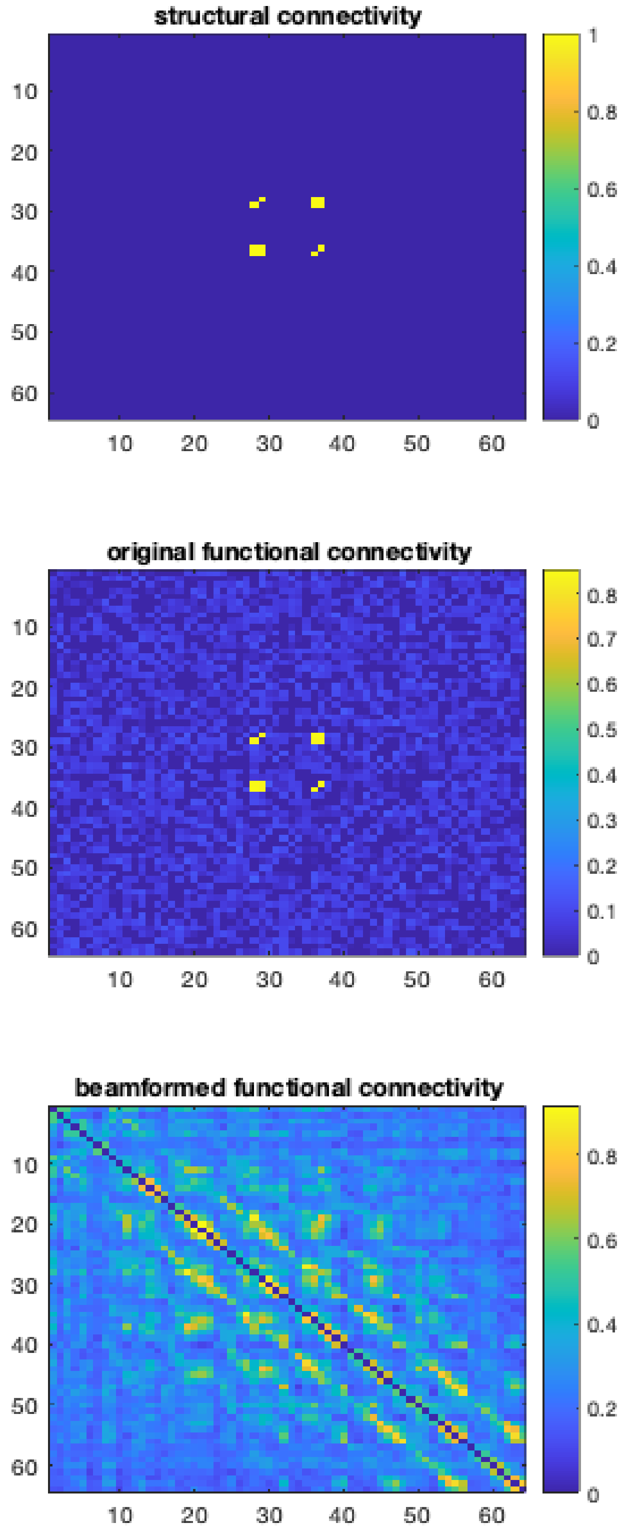

Supplementary Figure 20: Plots of Functional Connectivity (original and beamformed) and Structural Connectivity for 4 ictogenic nodes.

Node ictogenicity

Example 1: (Central)

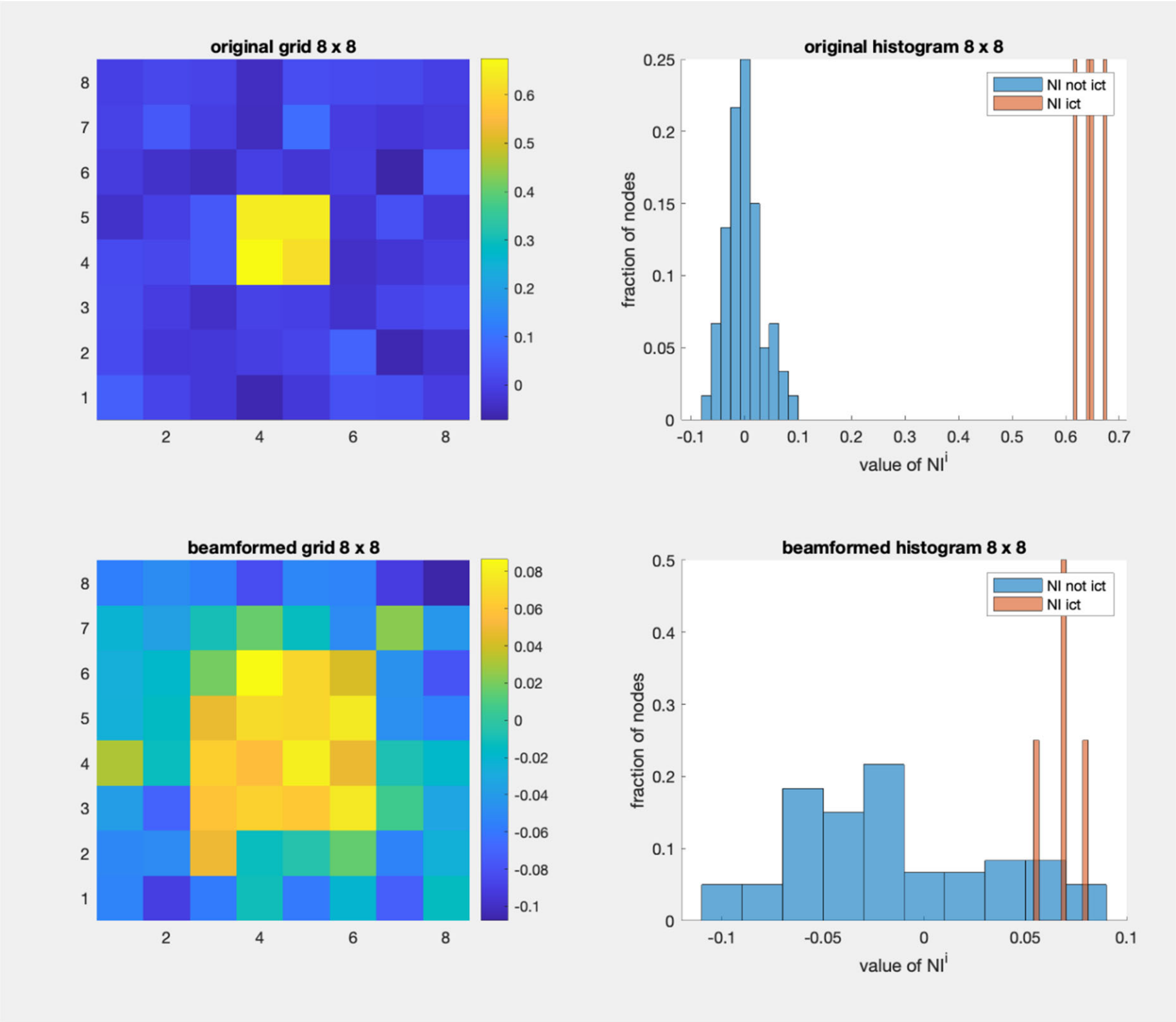

Supplementary Figure 21: Node Ictogenicity simulation for 4 central seizure generating nodes. The 8 x 8 grids (at left) correspond to node positions in the ViEEG grid. Yellow reflects high ictogenicity while dark blue reflects low ictogenicity. The histograms (at right) show the distributions of NI values for both ictogenic (orange) and non-ictogenic nodes (light blue). Abbreviations: ViEEG =virtual intracranial electroencephalography

The central 4 nodes are the seizure generating network. The functional connectivity network derived from the original time series can be used to calculate NI^i, which correctly identifies those nodes. The top left panel shows NI^ivalues for each of the nodes in the grid. The largest values are taken by the middle 4 nodes. After beamformer source reconstruction, the central nodes also carry high NI^i values, but so do some surrounding nodes, mainly due to the field leakage effect.

Example 2: (Upper Left)

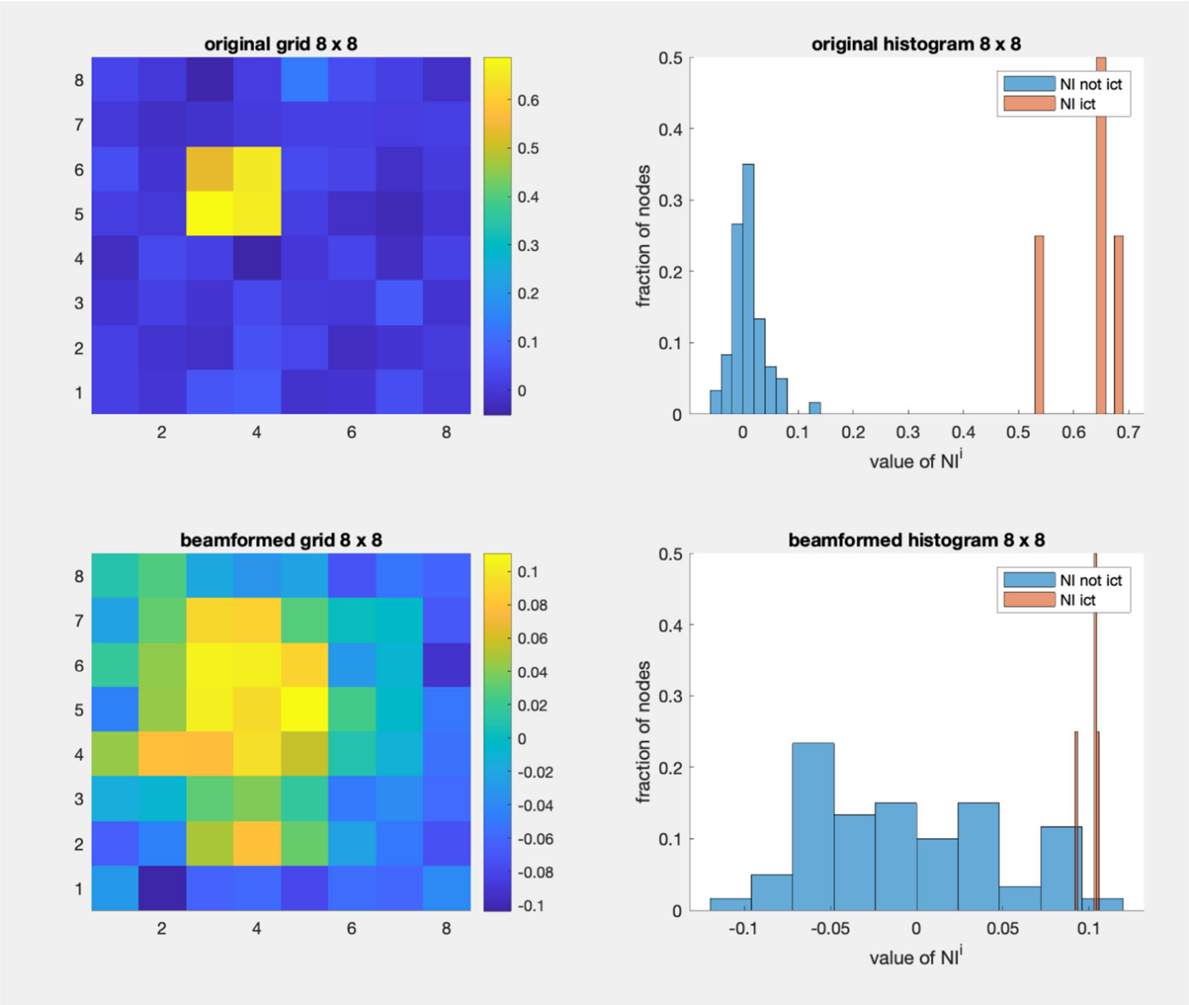

Supplementary Figure 22: Node Ictogenicity simulation results for 4 upper left seizure generating nodes.

As per the central example, the original functional connectivity can be used to identify the ictogenic nodes. Upon beamformer source reconstruction, a shift towards the upper left quadrant of the grid is observed, in the vicinity of the ictogenic nodes.

Example 3: (Lower Right)

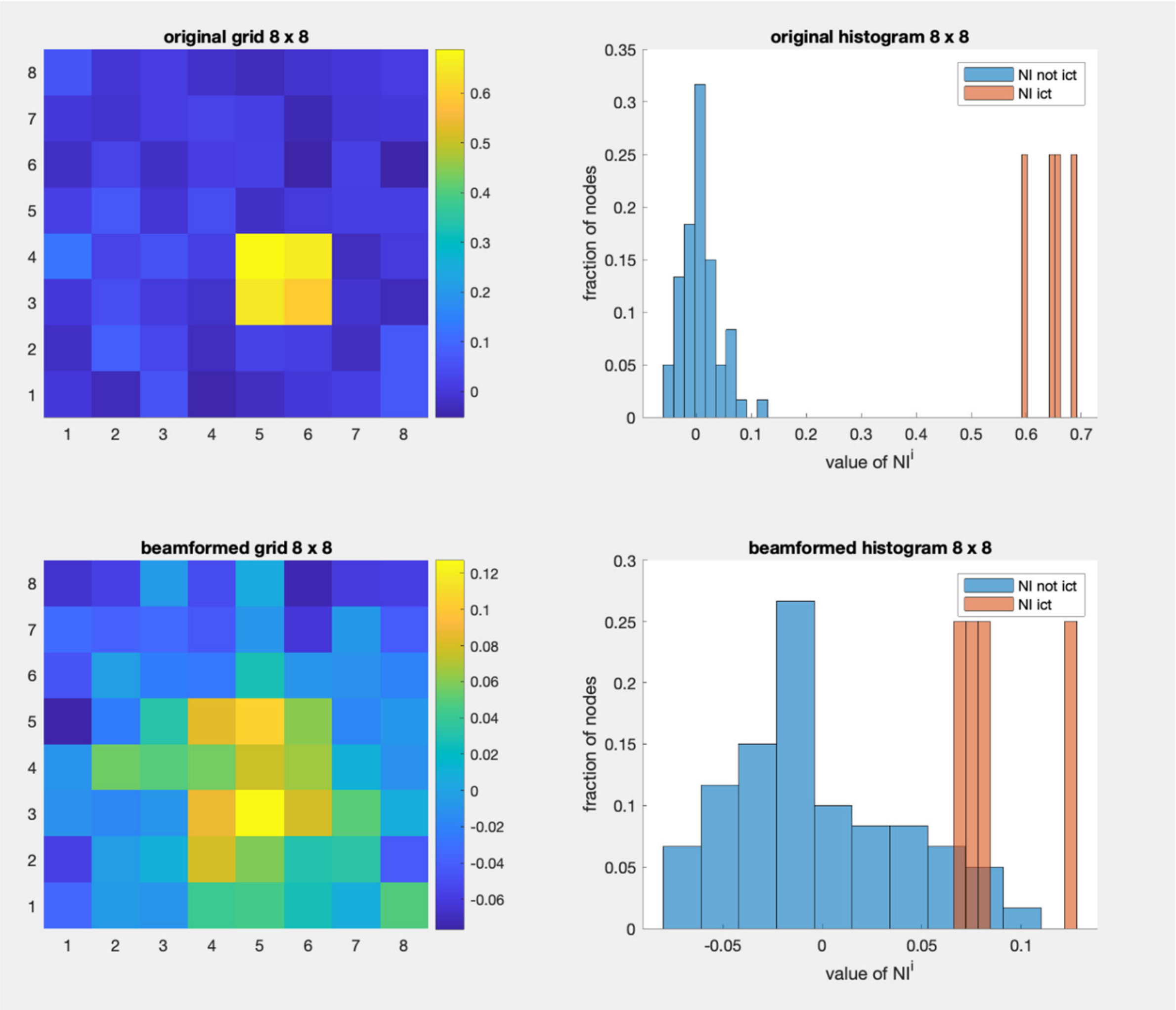

Supplementary Figure 23: Node Ictogenicity simulation results for 4 lower right seizure generating nodes.

Once again, after source reconstruction, the higher NI^i nodes for the beamformed grid overlap the re-positioned seizure generating nodes of the ViEEG grid.

References

1. Goodfellow M, Rummel C, Abela E, Richardson MP, Schindler K, Terry JR. Estimation of brain network ictogenicity predicts outcome from epilepsy surgery. *Sci Rep* **6**, 29215 (2016).

2. Lopes MA*, et al.* An optimal strategy for epilepsy surgery: Disruption of the rich-club? *PLoS Comput Biol* **13**, e1005637 (2017).

3. Lopes MA*, et al.* Elevated Ictal Brain Network Ictogenicity Enables Prediction of Optimal Seizure Control. *Front Neurol* **9**, 98 (2018).

4. Schreiber T, Schmitz A. Surrogate time series. *Physica D: Nonlinear Phenomena* **142**, 346-382 (2000).

5. Palva JM*, et al.* Ghost interactions in MEG/EEG source space: A note of caution on inter-areal coupling measures. *Neuroimage* **173**, 632-643 (2018).

6. Schoffelen JM, Gross J. Source connectivity analysis with MEG and EEG. *Hum Brain Mapp* **30**, 1857-1865 (2009).

7. Kraskov A, Stogbauer H, Grassberger P. Estimating mutual information. *Phys Rev E Stat Nonlin Soft Matter Phys* **69**, 066138 (2004).

8. Rummel C*, et al.* Resected Brain Tissue, Seizure Onset Zone and Quantitative EEG Measures: Towards Prediction of Post-Surgical Seizure Control. *PLoS One* **10**, e0141023 (2015).

9. Enatsu R*, et al.* Usefulness of MEG magnetometer for spike detection in patients with mesial temporal epileptic focus. *Neuroimage* **41**, 1206-1219 (2008).

10. Oishi M*, et al.* Fusiform gyrus epilepsy: the use of ictal magnetoencephalography - Case report. *Journal of Neurosurgery* **97**, 200-204 (2002).

11. Plummer C, Vogrin SJ, Woods WP, Murphy MA, Cook MJ, Liley DTJ. Interictal and ictal source localization for epilepsy surgery using high-density EEG with MEG: a prospective long-term study. *Brain* **142**, 932-951 (2019).

12. Ruzich E, Crespo-Garcia M, Dalal SS, Schneiderman JF. Characterizing hippocampal dynamics with MEG: A systematic review and evidence-based guidelines. *Hum Brain Mapp* **40**, 1353-1375 (2019).

13. Brookes MJ*, et al.* A general linear model for MEG beamformer imaging. *Neuroimage* **23**, 936-946 (2004).

14. Handy TC. *Brain Signal Analysis: Advances in Neuroelectric and Neuromagnetic Methods*. The MIT Press (2009).

15. Van Veen BD, van Drongelen W, Yuchtman M, Suzuki A. Localization of brain electrical activity via linearly constrained minimum variance spatial filtering. *IEEE Trans Biomed Eng* **44**, 867-880 (1997).

16. Sekihara K, Hild KE, 2nd, Nagarajan SS. A novel adaptive beamformer for MEG source reconstruction effective when large background brain activities exist. *IEEE Trans Biomed Eng* **53**, 1755-1764 (2006).

17. Sekihara K, Nagarajan SS, Poeppel D, Marantz A. Asymptotic SNR of scalar and vector minimum-variance beamformers for neuromagnetic source reconstruction. *IEEE Trans Biomed Eng* **51**, 1726-1734 (2004).

18. Hillebrand A, Singh KD, Holliday IE, Furlong PL, Barnes GR. A new approach to neuroimaging with magnetoencephalography. *Hum Brain Mapp* **25**, 199-211 (2005).

19. Gramfort A*, et al.* MNE software for processing MEG and EEG data. *Neuroimage* **86**, 446-460 (2014).

20. Fischl B*, et al.* Whole brain segmentation: automated labeling of neuroanatomical structures in the human brain. *Neuron* **33**, 341-355 (2002).

21. Hincapie AS*, et al.* The impact of MEG source reconstruction method on source-space connectivity estimation: A comparison between minimum-norm solution and beamforming. *Neuroimage* **156**, 29-42 (2017).

22. Colclough GL, Woolrich MW, Tewarie PK, Brookes MJ, Quinn AJ, Smith SM. How reliable are MEG resting-state connectivity metrics? *Neuroimage* **138**, 284-293 (2016).

23. Cosandier-Rimélé D, Ramantani G, Zentner J, Schulze-Bonhage A, Dümpelmann M. A realistic multimodal modeling approach for the evaluation of distributed source analysis: application to sLORETA. *Journal of Neural Engineering* **14**, 056008 (2017).

24. Akaike H. A new look at the statistical model identification. *IEEE Trans Automat Contr* **19**, 716-723 (1974).

25. Schwarz G. Estimating the Dimension of a Model. *Ann Stat* **6**, 461-464 (1978).
