# Supplementary material for: Virtual intracranial EEG signals reconstructed from MEG with potential for epilepsy surgery
Source: Nat Commun. 2022 Feb 22;13:994. doi: 10.1038/s41467-022-28640-x (PMC8863890; doi:10.1038/s41467-022-28640-x)
Supplement: Supplementary file 3 — Reporting Summary [file 41467_2022_28640_MOESM3_ESM.pdf]

nature research

Corresponding author(s): Chris Plummer
Last updated by author(s): Dec 19, 2021

Reporting Summary

Nature Research wishes to improve the reproducibility of the work that we publish. This form provides structure for consistency and transparency in reporting. For further information on Nature Research policies, see our Editorial Policies and the Editorial Policy Checklist.

Statistics

For all statistical analyses, confirm that the following items are present in the figure legend, table legend, main text, or Methods section.

n/a	Confirmed	
☐	☒	The exact sample size (n) for each experimental group/condition, given as a discrete number and unit of measurement
☐	☒	A statement on whether measurements were taken from distinct samples or whether the same sample was measured repeatedly
☐	☒	The statistical test(s) used AND whether they are one- or two-sided<br>*Only common tests should be described solely by name; describe more complex techniques in the Methods section.*
☐	☒	A description of all covariates tested
☐	☒	A description of any assumptions or corrections, such as tests of normality and adjustment for multiple comparisons
☐	☒	A full description of the statistical parameters including central tendency (e.g. means) or other basic estimates (e.g. regression coefficient) AND variation (e.g. standard deviation) or associated estimates of uncertainty (e.g. confidence intervals)
☐	☒	For null hypothesis testing, the test statistic (e.g. F, t, r) with confidence intervals, effect sizes, degrees of freedom and P value noted<br>*Give P values as exact values whenever suitable.*
☒	☐	For Bayesian analysis, information on the choice of priors and Markov chain Monte Carlo settings
☒	☐	For hierarchical and complex designs, identification of the appropriate level for tests and full reporting of outcomes
☒	☐	Estimates of effect sizes (e.g. Cohen's d, Pearson's r), indicating how they were calculated

Our web collection on statistics for biologists contains articles on many of the points above.

Software and code

Policy information about availability of computer code

Data collection	Curry 7® (Compumedics Neuroscan®), Elekta® Neuromag TRIUX Data Acquisition Software Release 6.0
Data analysis	Curry 8® (Compumedics Neuroscan®), Freesurfer 6, MNE-Python Toolbox, Python-based customized code, Stata 16.1, R 3.6

For manuscripts utilizing custom algorithms or software that are central to the research but not yet described in published literature, software must be made available to editors and reviewers. We strongly encourage code deposition in a community repository (e.g. GitHub). See the Nature Research guidelines for submitting code & software for further information.

Data

Policy information about availability of data

All manuscripts must include a data availability statement. This statement should provide the following information, where applicable:

- Accession codes, unique identifiers, or web links for publicly available datasets
- A list of figures that have associated raw data
- A description of any restrictions on data availability

All data are available upon request.

Field-specific reporting

Please select the one below that is the best fit for your research. If you are not sure, read the appropriate sections before making your selection.

☒ Life sciences ☐ Behavioural & social sciences ☐ Ecological, evolutionary & environmental sciences

For a reference copy of the document with all sections, see nature.com/documents/nr-reporting-summary-flat.pdf

Life sciences study design

All studies must disclose on these points even when the disclosure is negative.

Sample size	This is an extension of our earlier reported prospective study on 13 patients, 12 of whom had a total of 36 seizures captured by magnetoencephalography. All 12 patients were included in this ictal study and all patients had a minimum 2-year post-operative follow-up.
Data exclusions	As above, one patient did not have a seizure during the MEG recording and so was excluded from the present study. Source reconstructed epochs without clear ictal waveforms were excluded from the analysis (11 seizures). Therefore, 25 seizures from 12 patients were ultimately analysed.
Replication	Two network measures (amplitude envelope correlation and mutual information) were employed to verify stability of the ictogenic solutions provided by dynamical network models. We also used a sophisticated surrogate correction approach in constructing the networks.
Randomization	No retrospective randomization was applied because this is the follow-up study on our original cohort.
Blinding	Virtual intracranial electroencephalographic signals were source reconstructed without any clinical information. Dynamical network modelling was conducted using data without any accompanying clinical information.

Reporting for specific materials, systems and methods

We require information from authors about some types of materials, experimental systems and methods used in many studies. Here, indicate whether each material, system or method listed is relevant to your study. If you are not sure if a list item applies to your research, read the appropriate section before selecting a response.

Materials & experimental systems

n/a	Involved in the study	
☒	☐	Antibodies
☒	☐	Eukaryotic cell lines
☒	☐	Palaeontology and archaeology
☒	☐	Animals and other organisms
☐	☒	Human research participants
☒	☐	Clinical data
☒	☐	Dual use research of concern

Methods

n/a	Involved in the study	
☒	☐	ChIP-seq
☒	☐	Flow cytometry
☐	☒	MRI-based neuroimaging

Human research participants

Policy information about studies involving human research participants

Population characteristics	Thirteen patients (seven males, six females, age range 10-54 years, median 33 years; disease duration 3-32 years) had drug-resistant focal epilepsy with either no visible MRI lesion (10 patients) or a complex lesion (3 patients) and went on to resective surgery.
Recruitment	This retrospective analysis is an extension of our original study that consecutively recruited thirteen patients undergoing epilepsy surgery workup at Melbourne tertiary epilepsy centers.
Ethics oversight	The Human Research Ethics Committees of St Vincent's Hospital (ID: 34/08) and Swinburne University of Technology (ID: 2013/030), Melbourne

Note that full information on the approval of the study protocol must also be provided in the manuscript.

Magnetic resonance imaging

Experimental design

Design type	A single time point structural MRI scan prior to resective surgery was used to construct individualized head models for

Design type	source signal reconstruction.
Design specifications	A single time point MRI scan prior to resective surgery.
Behavioral performance measures	Not applicable.

Acquisition

Imaging type(s)	Structural
Field strength	3 Tesla
Sequence & imaging parameters	MPRAGE 1mm isotropic 3D acquisition, 256 axial slices, 256×256 matrix, Field of View 256×256mm, Echo Time = 1.97ms, Repetition Time = 2200ms, Inversion Time =900ms, flip angle degrees = 8.0, Number of averages = 1, using a 32-channel head coil
Area of acquisition	Whole brain

Diffusion MRI ☐ Used ☒ Not used

Preprocessing

Preprocessing software	Freesurfer 6 version 6.0.0
Normalization	MRI scans were not spatially normalized to a existing template as individualized head models were constructed from the native scans.
Normalization template	Not applicable.
Noise and artifact removal	B1 field intensity normalization was performed using N4 (standard Freesurfer 6 preprocessing steps)
Volume censoring	Not applicable.

Statistical modeling & inference

Model type and settings	Not applicable.
Effect(s) tested	Not applicable.

Specify type of analysis: ☒ Whole brain ☐ ROI-based ☐ Both

Statistic type for inference (See Eklund et al. 2016)	Not applicable.
Correction	Not applicable.

Models & analysis

n/a	Involved in the study
☒	☐ Functional and/or effective connectivity
☒	☐ Graph analysis
☒	☐ Multivariate modeling or predictive analysis
